# Two-Step Activation Mechanism of the ClpB Disaggregase for Sequential Substrate Threading by the Main ATPase Motor

**DOI:** 10.1016/j.celrep.2019.05.075

**Published:** 2019-06-18

**Authors:** Célia Deville, Kamila Franke, Axel Mogk, Bernd Bukau, Helen R. Saibil

**Affiliations:** 1 Department of Crystallography, Institute of Structural and Molecular Biology, Birkbeck, University of London, Malet Street, London WC1E 7HX, UK; 2 Center for Molecular Biology of University of Heidelberg (ZMBH) and German Cancer Research Center (DKFZ), DKFZ-ZMBH Alliance, Im Neuenheimer Feld 282, 69120 Heidelberg, Germany

**Keywords:** protein disaggregation, protein unfolding, AAA+, Hsp100, chaperone, cryo-EM

## Abstract

AAA+ proteins form asymmetric hexameric rings that hydrolyze ATP and thread substrate proteins through a central channel via mobile substrate-binding pore loops. Understanding how ATPase and threading activities are regulated and intertwined is key to understanding the AAA+ protein mechanism. We studied the disaggregase ClpB, which contains tandem ATPase domains (AAA1, AAA2) and shifts between low and high ATPase and threading activities. Coiled-coil M-domains repress ClpB activity by encircling the AAA1 ring. Here, we determine the mechanism of ClpB activation by comparing ATPase mechanisms and cryo-EM structures of ClpB wild-type and a constitutively active ClpB M-domain mutant. We show that ClpB activation reduces ATPase cooperativity and induces a sequential mode of ATP hydrolysis in the AAA2 ring, the main ATPase motor. AAA1 and AAA2 rings do not work synchronously but in alternating cycles. This ensures high grip, enabling substrate threading via a processive, rope-climbing mechanism.

## Highlights

• Multiple structures of ClpB disaggregase map out sequential firing of AAA domains
• ATP hydrolysis and exchange in the AAA2 domain drive protein translocation
• Offset firing of AAA1 and AAA2 maintains a constant grip on the substrate
• Docking of regulatory M-domains restrains ClpB dynamics and slows down threading

## Graphical Abstract

**Figure:**
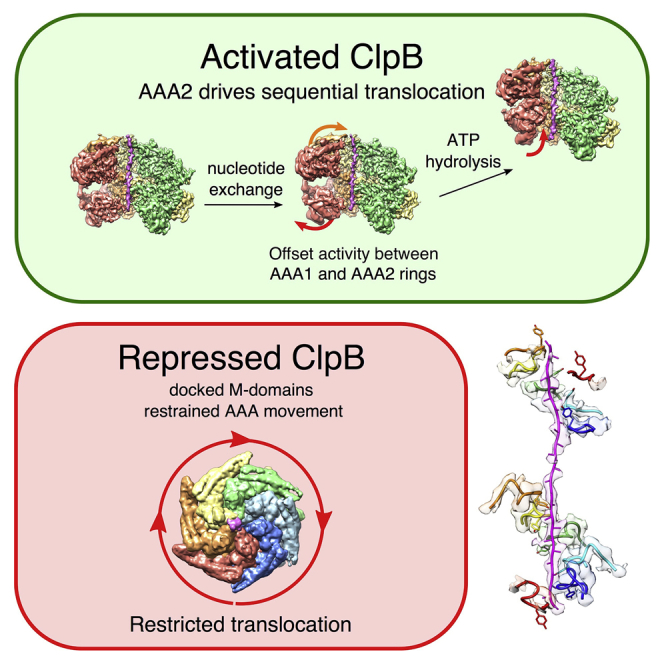

## Teaser

ClpB is a ring-shaped protein threading machine involved in protein disaggregation, regulated by an encircling ring of coiled-coil M-domains repressing the ATPase domains. Deville et al. determined a set of structures showing how ClpB activation by M-domain release triggers a sequential ATPase activity that translocates the polypeptide substrate.

## Introduction

AAA+ proteins couple energy from ATP hydrolysis to mechanical work, which is typically a directional threading activity linked to force generation for unwinding DNA or RNA, protein complex disassembly, protein unfolding, or protein disaggregation. They are usually hexamers, which can consist of a single layer of ATPase (AAA) domains or two tiers of tandem ATPase domains that form a ring-shaped oligomer with a central pore. Although the AAA+ superfamily is very large and diverse, common features are emerging for the core ATPase and threading mechanism.

For helicases, a sequential mechanism of ATP hydrolysis and substrate threading was proposed on the basis of crystal structures of DNA and RNA helicases (Enemark and Joshua-Tor, 2006, Itsathitphaisarn et al., 2012, Mancini et al., 2004, Moffitt et al., 2009, Thomsen and Berger, 2009, Thomsen et al., 2016). The ordered cycling of subunits between active and inactive states is propelled by ATP hydrolysis, which leads to stepwise transport of the substrate through the central channel.

Recently, a similar mode of ATPase and threading mechanism has been suggested for protein threading AAA+ members on the basis of structural snapshots of both single and tandem AAA domain complexes, several with model substrates bound in the central channel (de la Peña et al., 2018, Deville et al., 2017, Gates et al., 2017, Monroe et al., 2017, Puchades et al., 2017, Ripstein et al., 2017, Su et al., 2017, Wehmer et al., 2017, Yu et al., 2018, Zehr et al., 2017). Except for the ATPase ring of the proteasome regulatory subunit, these machines are homo-hexamers with a markedly asymmetric structure. The rings have varying degrees of spiral distortion, share one wider subunit interface (seam), and all harbor AAA domains in diverse structural states. Each AAA domain extends a flexible pore loop bearing a conserved aromatic residue, in most cases a tyrosine, which interacts with the backbone of the threading polypeptide. The pore loops are arranged to form a spiral staircase around the translocation channel. The presence of AAA domains in inactive and active states in the hexameric assemblies is consistent with a sequential mode of ATP hydrolysis and threading. Cycling of AAA domains between different activity states was recently observed directly, in case of the AAA+ rings of the homohexameric archaeal PAN (Majumder et al. 2019) and heterohexameric eukaryotic 26S proteasome regulatory subunit (de la Peña et al., 2018, Dong et al., 2019). However, it is unclear how ATPase and threading activities are coordinated in double-ring AAA+ hexamers composed of tandem AAA domains. How do the two AAA rings communicate, and what is the consequence for the threading mechanism? These questions relate directly to the reasons why some AAA+ proteins have two ATPase rings.

Furthermore, the reported structures do not explain the diversity of AAA+ protein activities, which vary considerably in threading processivity. For instance, the bacterial disaggregase ClpB exhibits lower processivity and unfolding power than its close homologs ClpA and ClpC, which work together with the peptidase ClpP in proteolysis (Haslberger et al., 2008, Li et al., 2015). The molecular basis of differing substrate threading processivities is unknown. In particular, the available structures do not address how the proposed sequential ATPase and threading mode are regulated. This is biologically significant as the activities of many AAA+ proteins are tightly controlled by partner proteins (adapters) and substrates, which typically stimulate ATP hydrolysis strongly (Davies et al., 2010, Davies et al., 2014, Lee et al., 2013, Rosenzweig et al., 2013, Schlothauer et al., 2003, Seyffer et al., 2012, Thomsen et al., 2016). Regulatory action restricts high AAA+ protein activity to the substrate-engaged state. The mechanistic details of this activation step and how it is linked to changes in AAA domain coordination and threading are largely unknown.

The disaggregase ClpB is a suitable model system to analyze control of ATPase and threading activities. ClpB activation requires two signals: (1) interaction with the Hsp70 partner chaperone and (2) binding to substrate protein (Lee et al., 2013, Oguchi et al., 2012, Rosenzweig et al., 2013, Seyffer et al., 2012). ClpB is composed of tandem AAA domains (AAA1, AAA2), an N-terminal domain (NTD), and a coiled-coil regulatory M-domain, which forms a repressive belt surrounding the AAA1 ring (Carroni et al., 2014, Heuck et al., 2016, Oguchi et al., 2012). Hsp70 binds to M-domains if they are released from their head-to-tail contacts, thereby derepressing ClpB. Full activation of the ClpB ATPase requires substrate binding as a second stimulus. Notably, ClpB activation by Hsp70 seems transient, as ClpB wild-type (WT) exhibits lower unfolding power during protein disaggregation than ClpB M-domain mutants that weaken M-domain interactions with the AAA1 ring to cause constitutive derepression (Haslberger et al., 2008, Oguchi et al., 2012). Such derepressed ClpB mutants are toxic *in vivo*, indicating that tight ClpB regulation is essential (Lipińska et al., 2013, Oguchi et al., 2012, Schirmer et al., 2004). The mechanistic basis of ClpB activation remains largely unknown. How do ATPase and threading activities differ between repressed and activated states?

Here we analyze ClpB activation by a combined biochemical and structural approach. We dissect the modes of substrate-stimulated ATP hydrolysis and compare the structures of substrate-engaged ClpB-WT and a constitutively derepressed M-domain mutant. A set of structural snapshots of the activated M-domain mutant strongly supports a sequential mechanism of ATP hydrolysis and substrate handover moving counterclockwise around the AAA2 ring that constitutes the main ATPase motor. Accordingly, AAA domains of the substrate-activated ClpB M-domain mutant hydrolyze ATP with reduced cooperativity. The structures also suggest that the regulatory AAA1 ring runs in a sequential mode, out of synchrony with the AAA2 ring, so that release and engagement of the substrate are anti-correlated between the rings. Such progressive cycling of AAA domains between active and inactive states is not observed for ClpB-WT with fully engaged substrate, suggesting that activation of ClpB-WT is transient.

## Results

### Activation of ClpB Coincides with Decreased Cooperativity

**Figure 1:**
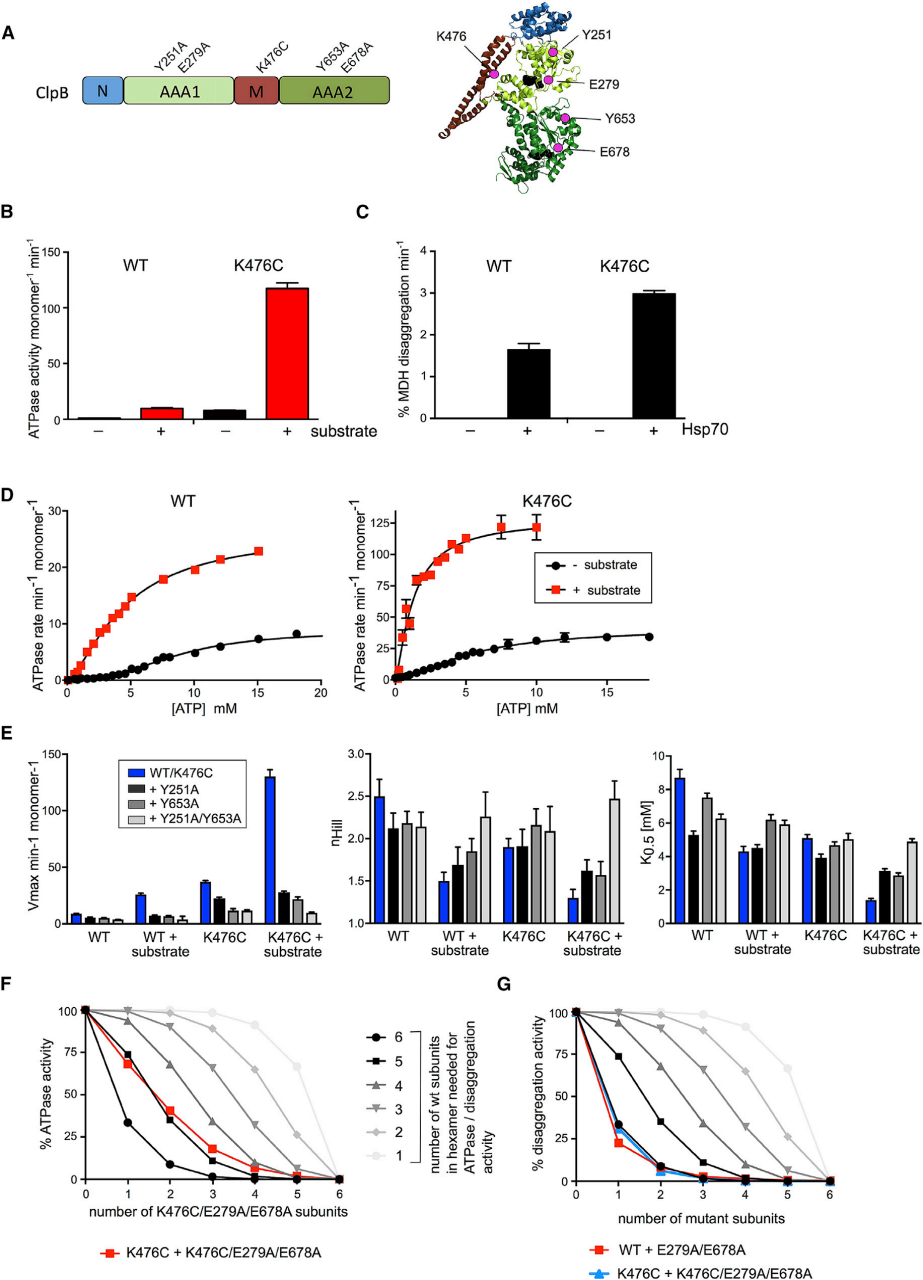
ClpB Activation Triggers a Sequential Mode of ATP Hydrolysis (A) ClpB domain organization and monomer structure. The identity and position of mutated residues are indicated. (B) ATPase activities of ClpB wild-type (WT) and ClpB-K476C were determined in the absence and presence of 10 μM casein (± substrate). SDs are indicated; for some points, error bars are shorter than the height of the symbol and are not depicted. (C) MDH disaggregation activities of ClpB-WT and ClpB-K476C in the absence and presence of Hsp70. (D) ATPase activity of ClpB-WT and ClpB-K476C in absence and presence of casein (± substrate) as a function of ATP concentration. (E) v_max_ of ATPase activities, derived Hill coefficient (h), and ATP concentrations at half-maximal ATPase activity (*K*_0.5_) for WT, pore 1 (Y251A), and pore 2 (Y653A) loop mutants of ClpB-WT and ClpB-K476C. (F and G) ATPase activities of ClpB-K476C/ClpB-K476C/E279A/E678A (F) and MDH disaggregation of ClpB-WT/ClpB-E279A/E678A (G) mixes were determined (red, blue). They are compared with curves calculated from a model (black to gray) that assumes that a mixed hexamer only displays ATPase or disaggregation activity if it contains the number of wild-type subunits indicated. Mixing ratios are indicated as number of E279A/E678A mutant subunits.

We sought to biochemically and structurally dissect the Hsp70 and substrate-dependent process of ClpB activation. The interaction between Hsp70 and ClpB is transient, hampering analysis of an Hsp70-bound state of ClpB. We therefore made use of the derepressed ClpB-K476C M-domain mutant, which mimics the transient state of ClpB activation by Hsp70 (Oguchi et al., 2012). Lys476 is part of a conserved salt bridge network that regulates the dynamic interaction between M-domain and AAA1 ring (Figure 1A) (Lipińska et al., 2013, Oguchi et al., 2012). This interaction is weakened in ClpB-K476C, resulting in M-domain dissociation and persistent, Hsp70-independent derepression of ClpB ATPase activity. Consequently, ATPase activation by substrate is much stronger than in ClpB-WT (Figure 1B; Table S1), and ClpB-K476C has increased protein disaggregation activity (Figure 1C), linked to its ability to unfold stable domains, an activity not observed for ClpB-WT (Oguchi et al., 2012). Because high ATPase activity of ClpB-K476C requires substrate binding, we used the disordered model substrate casein, which is directly recognized by ClpB, to study the activation process.

Both basal and substrate-induced steady-state ATPase activities of ClpB-K476C are strongly increased over those of ClpB-WT. ClpB-WT and ClpB-K476C do not differ in nucleotide affinities (Franke et al., 2017), raising the possibility that an increased affinity of ClpB-K476C for casein might explain stronger stimulation of ATPase activity. We first determined ClpB-WT and ClpB-K476C ATPase activities at increasing substrate concentrations. Casein concentrations at half-maximal ATPase activities were comparable for ClpB-WT and ClpB-K476C (3.6 ± 0.5 versus 3.1 ± 0.4 μM) (Figure S1A), suggesting similar substrate affinities. This was confirmed by determining similar binding affinities of ClpB-WT and ClpB-K476C for fluorescein isothiocyanate (FITC)-casein in fluorescence anisotropy experiments (*K*_d_ = 0.33 ± 0.05 versus 0.22 ± 0.04 μM) (Figure S1B). These findings exclude differences in substrate affinity as the basis for the enhanced ATPase activity of ClpB-K476C. We therefore determined ATPase activities at increasing ATP concentrations to analyze whether altered communication between ATPase subunits (e.g., increased cooperativity) causes the differing ATPase activities. Basal ATPase activity of ClpB-WT showed a sigmoidal curve, indicating a cooperative mode of ATP hydrolysis (Figure 1D). The determined Hill coefficient (h = 2.5 ± 0.2) agrees well with values determined for *T. thermophilus* ClpB (h = 2.7) (Schlee et al., 2001) and the yeast homolog Hsp104 (h = 2.3) (Hattendorf and Lindquist, 2002). Notably, casein addition reduced the Hill coefficient of ClpB-WT (h = 1.5 ± 0.1) and that of ClpB-K476C even more (h = 1.3 ± 0.1) (Figures 1D and 1E; Table S1), to almost Michaelis-Menten-like ATPase kinetics, with little positive cooperativity. Furthermore, ATP concentrations at half-maximal ATP hydrolysis rates of ClpB-K476C dropped to 1.4 mM in the presence of substrate, compared with 4.3 mM for WT ClpB in the presence of substrate. This indicates that only the fully two-step activated state of ClpB reaches high ATPase activity at physiological ATP concentrations (Figures 1D and 1E; Table S1), which coincides with decreased cooperativity.

To substantiate the substrate-triggered decrease in cooperativity of ClpB-K476C ATPase activity, we determined the ATPase parameters of ClpB-K476C pore loop mutants (AAA1 loop Y251A, AAA2 loop Y653A) (Figures 1A and 1E; Figure S1C), which exhibit defects in substrate interaction (Deville et al., 2017, Lum et al., 2004, Weibezahn et al., 2004). Pore loop mutations increased Hill coefficients in the presence of substrate, and the double pore loop mutations ClpB-K476C-Y251A-Y653A restored WT-like cooperativity of ATP hydrolysis (h = 2.47 ± 0.21) (Figure 1E; Table S1). These findings imply that substrate binding changes the mode of ATP hydrolysis in the ClpB-K476C hexamer, reducing positive cooperativity.

A reduction in ATPase cooperativity upon ClpB activation is consistent with a sequential mode of ATP hydrolysis, which has been proposed for various AAA+ protein unfoldases (de la Peña et al., 2018, Dong et al., 2019, Gates et al., 2017, Majumder et al., 2019, Monroe et al., 2017, Puchades et al., 2017, Ripstein et al., 2017), on the basis of cryoelectron microscopy (cryo-EM) structures. A sequential mechanism requires subunit coordination for successive ATP hydrolysis by adjacent ClpB subunits. A particularly high degree of subunit coordination is therefore expected for derepressed ClpB-K476C in the presence of substrate. We tested this prediction by determining ATPase activities of mixed hexamers composed of ClpB-WT or ClpB-K476C and the corresponding ATPase-deficient E279A/E678A (double Walker B [DWB]) mutant subunits. Only the substrate-stimulated ClpB-K476C ATPase activity was reduced upon incorporation of mutant subunits, confirming increased subunit coordination (Figures S2A and S2B; Kummer et al., 2016). Notably, the presence of catalytically dead DWB subunits increased ATP hydrolysis in the active subunits of mixed oligomers in case of ClpB-WT (without and with substrate) and for ClpB-K476C in the absence of substrate, relative to ClpB-WT controls (Figure S2B). This can be explained by altered intra- or inter-ring communications between AAA domains and indicates that introducing some AAA domains locked in an ATP state can trigger ATP hydrolysis in the others. Strikingly, this regulatory mode is not observed for fully activated ClpB mimicked by substrate-bound ClpB-K476C, whose ATPase activity is poisoned by mutant subunit incorporation (Figure S2A). The degree of ClpB-K476C ATPase poisoning was reduced when adding ClpB-K476C-DWB pore loop mutants (Y251A, Y653A) in mixing experiments (Figure S2C), underlining the impact of substrate binding in changing the mode of ATP hydrolysis.

We calculated the number of DWB subunits required to block ATP hydrolysis in a ClpB-K476C hexamer (+ casein). We compared the measured ATPase activities with those derived from a model assuming that a mixed hexamer displays activity only if it contains a certain number of WT subunits (Figure 1E). We found that incorporation of two DWB subunits abrogated the substrate-induced ATPase activity of ClpB-K476C. We considered this number a potential overestimate given the observed increase in activity of ClpB-K476C ATPase proficient subunits in the absence of substrate upon DWB incorporation. Thus the presence of even a single DWB subunit might be sufficient to block the substrate-induced high-ATPase activity mode of ClpB-K476C. This inhibition is also seen in a functional assay probing for disaggregation activities of mixed oligomers as the readout (Figure 1F). Here, the incorporation of a single DWB subunit is sufficient to abrogate disaggregation of aggregated malate dehydrogenase by ClpB-WT or ClpB-K476C. The high sensitivity of ClpB-WT can be explained by its transient activation upon binding the Hsp70 partner during disaggregation.

We conclude that the activated state of ClpB hydrolyses ATP with low positive cooperativity yet in a highly coordinated manner. This is consistent with a sequential mode of ATP hydrolysis, which is specifically initiated upon ClpB activation, involving dissociation of M-domains and substrate binding to the ClpB pore sites.

### Substrate-Bound ClpB-K476C Structures Reveal Large Displacements of AAA2 Pore Loops

**Figure 2:**
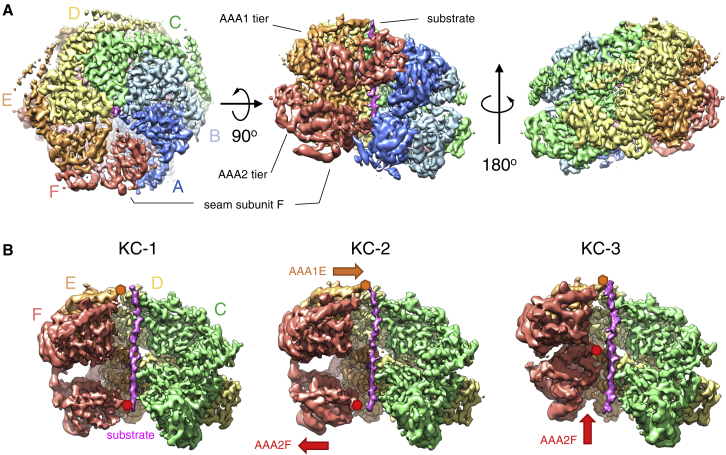
Overview of Substrate-Bound ClpB-DWB-K476C (A) Left, top view, and middle and right, side views of the cryo-EM density map of the most populated conformation of casein-bound ClpB-DWB-K476C (KC-2). The six protomers form a closed ring with a helical arrangement of two stacked AAA tiers and a seam between subunits A and F. The flexible N-terminal domains, located above the AAA1 tier, are not visible at high contour level. M-domains are partly visible for protomers C–E. (B) Views of the cryo-EM maps of the three states of substrate-bound ClpB-DWB-K476C. Densities of protomers A and B are removed to show conformational changes in protomers AAA1E and AAA2F, highlighted by orange and red arrows, respectively. Orange and red hexagons show the position of moving AAA1E and AAA2F pore loops.

In order to characterize the structural basis of ClpB activation and the basis for the observed changes in cooperativity, we used single-particle cryo-EM to determine the structure of ClpB-K476C in its substrate-bound state. To allow stable substrate trapping, we used a ClpB-K476C-DWB variant and incubated it with an excess of casein in the presence of ATPγS. Substrate-bound ClpB-K476C protomers form a closed ring state and adopt an asymmetric arrangement with a right-handed spiral distortion closed by a seam between protomers A and F, as previously observed for ClpB-WT (Figure 2A; Deville et al., 2017, Yu et al., 2018). The map of substrate-bound ClpB-K476C refined to a resolution of 3.4 Å (Figures S3A–S3D; Table S3). Further three-dimensional (3D) classification allowed the identification of four states differing mainly in the positions of the seam subunits (Figure S4A). Those classes refined to resolutions between 3.6 and 4.1 Å. Even after extensive sorting by 3D classification, the seam protomers are less resolved than the rest (4.5–6 Å resolution versus 3.5–4 Å resolution; Figures S3A–S3D; Table S3), highlighting that this region represents a dynamic hotspot. The states were numbered according to the position of the AAA2 domain of the seam protomer F, the region undergoing the biggest conformational change and were termed KC-1, KC-2A/2B, and KC-3 (Figure 2B). KC-2A and KC-2B differ only partially in the orientation of AAA2F (Figures S5A and S5B) and show otherwise identical features; they were therefore defined as KC-2 and are described together unless specified otherwise. The states KC-1, KC-2, and KC-3 were populated to 16%, 33%, and 17%, respectively.

The substrate casein is bound over a distance of 75 Å, corresponding to 24 residues. The main contacts are provided by AAA1 and AAA2 pore loops containing the conserved tyrosines (Y251 and Y653) and a charged loop in AAA1 (L1′), as previously described (Deville et al., 2017). The pore loops stabilize casein in an extended conformation via hydrogen bonds between the backbones of K250, Y251 (AAA1), G652, and Y653 (AAA2) and the backbone of the substrate (Figures S5C and S5D). The helically stacked side chains of Y251 and R252/E256 in AAA1 and Y653 and V656 in AAA2 form pockets to accommodate side chain of the substrate (Figures S5C and S5D), as recently described for *M. tuberculosis* ClpB (Yu et al., 2018).

**Figure 3:**
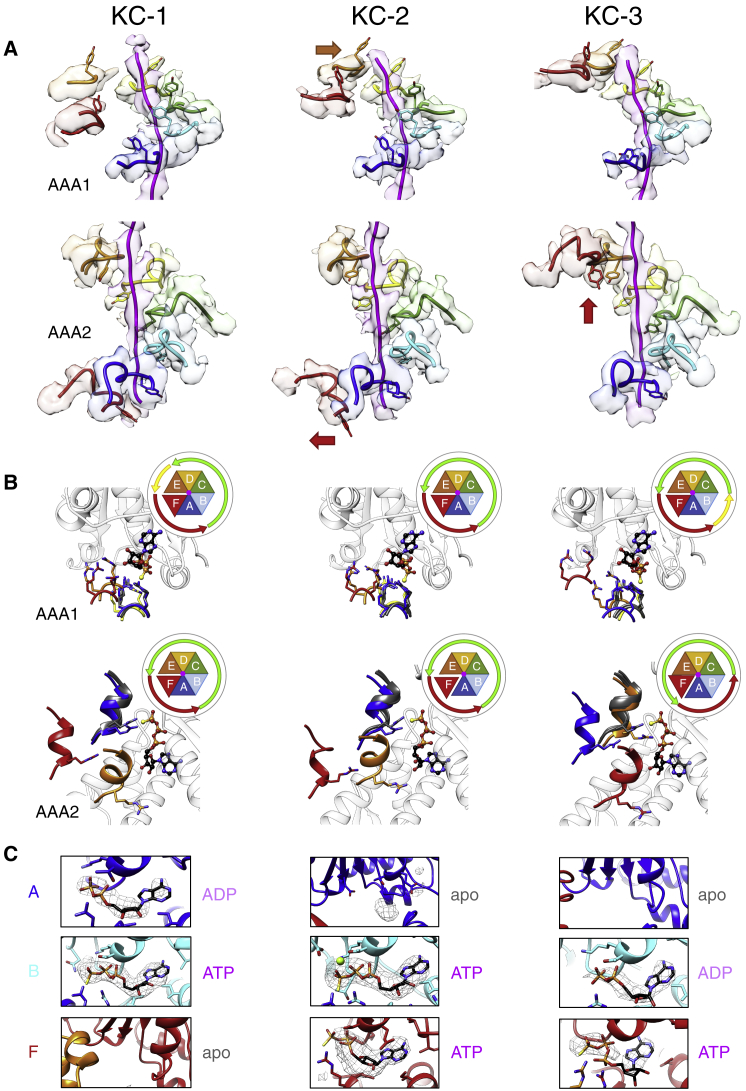
Pore Loop Movements and Arginine Finger Contacts in the Three States of Substrate-Bound ClpB-DWB-K476C Suggest a Sequential Mechanism of ATP Hydrolysis and Substrate Threading (A) Pore loop interactions with the substrate in AAA1 (top panels) and AAA2 (bottom panels) rings. The pore loop AAA1E (orange) engages the substrate in KC-2, while the pore loop AAA2F (red) dissociates. AAA2F moves from the bottom to the top of the staircase of pore loops in KC-3. (B) Arginine finger engagements in the AAA1 and AAA2 ring. All protomers were aligned to the large lobe of AAA1 or AAA2 domain of protomer C to compare engagement of the arginine fingers with neighboring subunits. Arginine fingers of AAA1B–C and AAA2B–D are shown as gray ribbons and interact with the γ-phosphate of ATP bound at the active site of a neighboring subunit in all three states. Activity states of AAA2 protomers are indicated by green (active) and red (inactive) arrows. (C) Nucleotide densities for AAA2A, AAA2B, and AAA2F protomers and assigned nucleotide state.

The interactions and positions of pore loops of individual protomers differ considerably between the different ClpB-K476C states, but their total number remains at ten. In state KC-1, all six AAA2 pore loops contact the substrate with the AAA2F protomer positioned at the bottom of the spiral staircase (Figure 3A, left panel). In the AAA1 ring, pore loops A–D are bound to the substrate, and protomers E and F are detached, with pore loop E at the top of the spiral arrangement of AAA1 loops and pore loop F in an intermediate position (Figure 3A, left panel). In state KC-2 the pore loop of AAA1E engages the substrate at the highest position, above AAA1D, while the pore loop of AAA2F is dissociated from the substrate at the lowest position (Figures 2B and 3A, middle panel). Substrate binding by pore loops is therefore anti-correlated in AAA1 (gain) and AAA2 (loss), thus maintaining a total of ten pore loops gripping the substrate.

The biggest displacement is observed for pore loop AAA2F, which moves upward by 34 Å, placing it at the top of the AAA2 pore loop track in state KC-3 (Figures 2B and 3A). The AAA2F pore loop is now placed to grab the substrate two residues above AAA2E (Figure 3A). Therefore, these three states of ClpB-K476C show sequential repositioning of pore loops around the ring with binding of AAA1E at the top of the spiral track of substrate interactions and cycling of AAA2F from the bottom to the top position of the AAA2 pore loops (Video S1).

Video S1 Detail of Pore Loop Movements across ClpB-K476C States and Sequential Translocation of a Polypeptide Extracted from an Aggregate in a Fully Activated ClpB, Related to Figure 3. A morph was created using KC-1, KC-2A (labeled KC-2 in the movie), KC-2B and KC-3 states. Substrate binding (2-residue translocation step) and release are out of step in AAA1 and AAA2 ensuring a constant grip on the substrate.

### Counterclockwise Cycling of AAA2 Domains between Active and Inactive States Indicates a Sequential Mode of ATP Hydrolysis upon ClpB Activation

We next analyzed whether the differences in pore loop positions between the ClpB-K476C structural states correlate with changes in the activity states of the respective AAA domains. To be active and competent for ATP hydrolysis, a AAA domain will have ATP bound while concurrently receiving a *trans*-acting arginine finger (AAA1: R331, R332; AAA2: R756), which contacts the γ-phosphate group of ATP, from the clockwise neighboring subunit (Biter et al., 2012, Yamasaki et al., 2011, Zeymer et al., 2014). We observe evidence for a counterclockwise change in the activity states of AAA domains in the AAA1 and AAA2 rings. Nucleotide exchange and hydrolysis events in AAA2 appear to drive the repositioning of the seam subunit AAA2F associated with a two-residue translocation step.

In all states, all AAA1 domains have full occupancy of ATPγS bound except AAA1F in KC-1 and KC-2 and AAA1A in KC-3, for which ADP was fitted into a weaker nucleotide density (Figure S6A). In all states, AAA1C and AAA1D can be defined as active in the AAA1 ring (ATP-bound and receiving arginine fingers of AAA1B and AAA1C, respectively), and AAA1A and AAA1F can be defined as inactive (displaced arginine fingers of AAA1F and AAA1E respectively, by 6.5–7 Å). However, the activity states of AAA1B and AAA1E vary (Figure 3B). In KC-1, the arginine finger of AAA1D is moderately displaced by 3 Å. This intermediate position suggests AAA1E is in an intermediate, not fully active state. In KC-2, substrate engagement by AAA1E is linked to AAA1E activation as the arginine finger of AAA1D fully moves in. In KC-3, displacement of the arginine finger of AAA1A by about 2 Å relative to active arginine fingers implies partial loss of AAA1B activity. Therefore, subtle conformational changes of arginine fingers in the AAA1 ring point toward a counterclockwise cycling of active subunits, including gain of activity (AAA1E in KC-2) and loss of activity (AAA1B in KC-3) (Figure 3B).

In case of the AAA2 ring we observe larger conformational changes. In KC-1 and KC-2, the nucleotide binding pockets of AAA2B–AAA2E show clear densities for ATPγS and receive arginine fingers of their counterclockwise neighbors (Figures 3B, 3C, and S6B), defining AAA2B – AAA2E as active. In contrast, the arginine fingers of AAA2E and AAA2F shift 12-16 Å away from the nucleotide sites of AAA2F and AAA2A, respectively, defining AAA2F and AAA2A as inactive. In KC-3, gain of activity of AAA2F and loss of activity of AAA2B leads to a counterclockwise cycling of the four active subunits by one protomer. This cycling across three states is linked to nucleotide exchange and likely hydrolysis at the seam (Figures 3B and 3C). AAA2F dissociation from the substrate coincides with nucleotide release from AAA2A and nucleotide binding to AAA2F (Figure S7). The lower resolution of the cryo-EM map at the seam subunit AAA2F does not allow unambiguous identification of the bound nucleotide, but we think it is likely to be ATPγS, as imposed by a sequential cycle of ATP hydrolysis. AAA2F activation is coupled with the upward movement of its pore loop, demonstrating tight linkage between the active sites providing and transmitting the energy derived from ATP hydrolysis.

How does ClpB ensure that only one out of four active AAA2 domains hydrolyses ATP at a time? In KC-3, the ADP nucleotide density in AAA2B and the displacement of neighboring AAA2A arginine finger by 7 Å from the nucleotide (Figures 3B and 3C) suggest that ATP hydrolysis at AAA2B coincides with conversion from KC-2 to KC-3 and AAA2B inactivation. In KC-2, an apo AAA2A provides an arginine finger to AAA2B, while all other clockwise neighbors of active subunits AAA2 C–E bind ATP. This suggests that the nucleotide content of an AAA2 domain determines ATPase activity of its counterclockwise neighbor, and an apo state triggers ATP hydrolysis in an active neighbor, explaining how ATP hydrolysis sequentially progresses around the AAA2 ring (Figures 3B and 3C).

**Figure 4:**
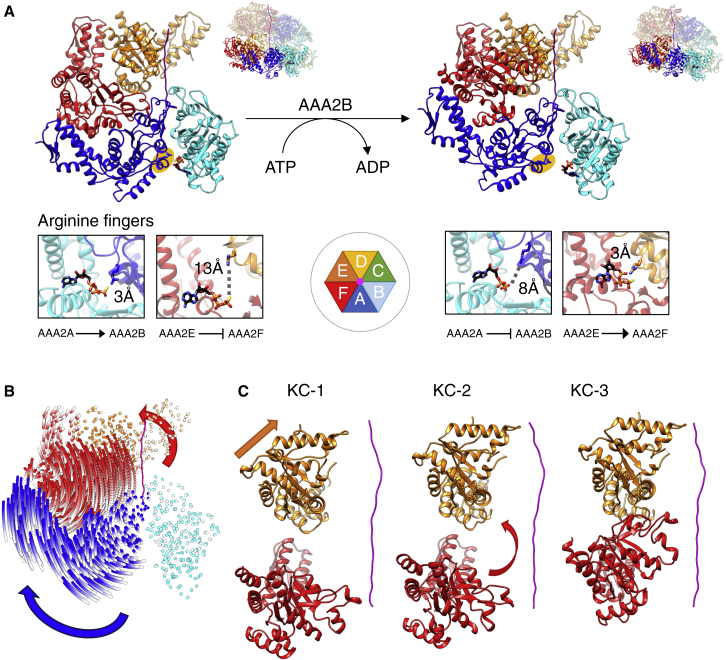
Activation and Inactivation of Subunits in the AAA2 Ring Are Directly Coupled (A) Views of the AAA2 domains of protomers A, B, E, and F for states KC-2 and KC-3 are shown. The small lobe of AAA2B is omitted for clarity. In state KC-2 (left), the arginine finger of AAA2A (highlighted by yellow oval) contacts the γ-phosphate of ATP bound at neighboring AAA2B. In the post-hydrolysis state KC-3 (right), detachment of this arginine finger allows rotation of AAA2A by 14° to move away from AAA2B while remaining bound to the substrate. This rotation is transmitted to AAA2F, causing its repositioning to the top of the spiral track of AAA2 pore loops and its activation by receiving an arginine finger from AAA2E. (B) The track of Cα atoms when morphing from KC-2 to KC-3 illustrates the amplitude of movements of AAA2A and AAA2F at the seam of the AAA2 ring. Blue and red arrows highlight the rotations of AAA2A and AAA2F subunits, respectively. (C) Activation of AAA1E is a prerequisite for AAA2F rotation. Views of AAA1E, AAA2F, and casein substrate in states KC-1, KC-2, and KC-3 are shown.

Inactivation of AAA2B and activation of AAA2F subunits are coupled via the interconnecting AAA2A subunit. ATP hydrolysis at AAA2B causes dissociation of the AAA2A arginine finger and rotation of AAA2A by 14° while it remains bound to the substrate. This movement is transmitted to the neighboring F protomer and causes a 44.5° rotation of AAA2F and an upward movement, allowing the arginine finger of AAA2E to contact the ATP bound at AAA2F (Figures 4A and 4B) and positioning of the AAA2F pore loop at the top of the spiral track of AAA2 loops.

Interconversions of AAA2 domains between the three states support a sequential mechanism of ATP hydrolysis (Video S1), in agreement with our biochemical analysis. Activation of the seam subunit AAA2F in state KC-3 is linked to resetting of its pore loop from the bottom to top position, indicating that sequential ATP hydrolysis around the ring dictates substrate threading in sequential, discrete steps on the substrate.

### Offset Cycling of AAA1 and AAA2 Rings

The comparison of structural changes in the ClpB-K476C AAA1 and AAA2 rings provides the first insights into ring coupling in tandem AAA+ proteins. Although we observe cycling of subunits in both AAA1 and AAA2 rings, this does not happen synchronously but with an offset of one subunit in different structural states (Figure 3B). This offset is also seen in the organization of the ClpB hexamer, in which the AAA1 domain of one subunit is positioned above the AAA2 domain of its counterclockwise neighbor. In state KC-2, AAA1E becomes active in the AAA1 ring while AAA2E is already active in KC-1. Similarly, activation of AAA2F in the AAA2 ring occurs in state KC-3, while AAA1F remains inactive. Thus, the AAA1 ring lags one counterclockwise subunit “behind” the AAA2 ring, and the changes in activity states do not happen simultaneously. Similarly, substrate engagement by the AAA1E pore loop in state KC-2 is coupled to substrate dissociation of the AAA2F pore loop (Figure 3A). Notably, activation of AAA1E in state KC-2 is linked to an upward movement, which creates the space necessary for the large rotation and upward movement of AAA2F upon conversion of state KC-2 to KC-3 (Figure 4C). Therefore, the offset cycling of the AAA1 ring seems prerequisite for sequential cycling of the AAA2 ring.

### The AAA2 Domain Constitutes the Main Threading Motor

Our structural analysis of ClpB-K476C shows that ClpB activation triggers sequential repositioning of pore loops. This is most pronounced for AAA2, suggesting that this domain represents the main motor of the disaggregase. We analyzed the roles of AAA1 and AAA2 domains upon ClpB activation by mutating key residues in ATP hydrolysis (Walker B motif: E279A, E678A; arginine finger: R331A, R756A) or substrate interaction (pore loop: Y251A, Y653A) in derepressed ClpB-K476C and tested their impact on disaggregation and ATPase activities (Figures S2A and S2D–S2G). Walker B and arginine finger mutations strongly reduced protein disaggregation activities for ClpB-WT and ClpB-K476C. This indicates that derepressed ClpB-K476C requires two functional AAA domains for efficient disaggregation. However, the AAA1 mutations (E279A, R331A) linked to ClpB-K476C retained more of the WT disaggregation activity than the corresponding AAA2 mutants (Figures S2D and S2E). A functional AAA2 domain is therefore essential for ClpB activation, whereas defects in AAA1 can be partially compensated by constitutive derepression (ClpB-K476C). This effect became particularly obvious when determining disaggregation activities of pore loop mutants. Pore loop 2 (Y653) was essential for disaggregation in ClpB-WT and ClpB-K476C. In contrast, the pore loop 1 (Y251A) mutant retained partial activity in ClpB-WT, which was further stimulated upon ClpB derepression to almost WT-like activity (Figures S2D and S2E). We infer that persistent ClpB activation partially compensates for AAA1 defects, while being strictly dependent on a functional AAA2 domain. Accordingly, blocking ATP hydrolysis at AAA2 in ClpB-K476C-E678A strongly reduced substrate-stimulated ATPase activity, while inhibiting AAA1 activity had a more modest effect (7.6-fold versus 1.7-fold reduction, respectively) (Figures S2F and S2G). These observations define the AAA2 domain as the main ATPase motor, which is particularly stimulated upon ClpB activation.

### Structures of Substrate-Bound ClpB-WT Do Not Show Coupled Cycling of AAA2 Domains

**Figure 5:**
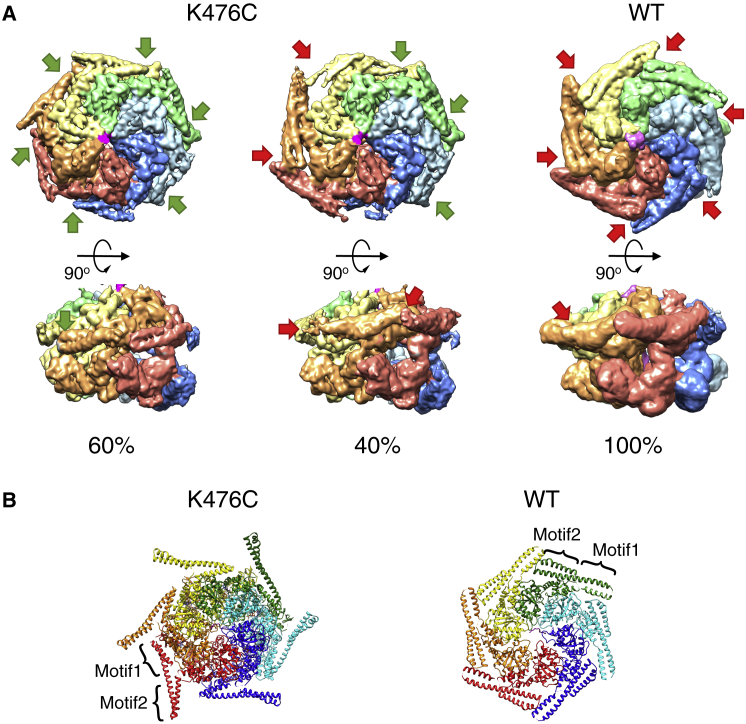
Docked M-Domains Repress the Activity of ClpB-WT and Reduce the Range of AAA Domain Movements (A) Heterogeneity of M-domain conformations. Top and side views of the cryo-EM density maps for the two main M-domain conformations of the ClpB-DWB-K476C:casein complex and for the ClpB-DWB:casein complex. Detached M-domains are indicated by green arrows and docked M-domains by red arrows. (B) Docking states of M-domains: atomic models showing the predominant conformation of M-domains enclosing the AAA1 tier in ClpB-K476C (left) and ClpB-WT (right) states. In ClpB-WT, M-domains are docked in a horizontal conformation that is stabilized by head-to-tail interactions between motif 1 and motif 2 of neighboring M-domains. In ClpB-K476C, M-domains adopt a tilted conformation with motif1 contacting the AAA1 domain of the adjacent protomer. Head-to-tail interactions are broken, rendering M-domain motif 2 invisible in the cryo-EM maps. Here, full-length M-domains are shown, docked in the density of motif1, to emphasize the differences in M-domain docking states between ClpB-WT and ClpB-K476C.

Our biochemical analysis pinpoints strong differences in ATPase activities of substrate-stimulated ClpB-WT and derepressed ClpB-K476C (Figure 1). To investigate the underlying mechanism we aimed at a direct structural comparison of substrate-bound ClpB-WT and ClpB-K476C. Our previous analysis of substrate-bound ClpB (BAP)-WT yielded a structure that is similar to KC-1 of ClpB-K476C, but the map was of lower resolution (4.5–5 Å) (Deville et al., 2017). To permit a better comparison, we took advantage of improved sample preparation methods and redetermined the structure of ClpB-DWB in complex with casein in the presence of ATPγS (Figures 5A and S3E–S3H; Table S3).

After two-dimensional (2D) classification, a map was obtained that refined to a resolution of 3.9 Å but still displayed a poorly resolved seam area and anisotropy because of preferential end view orientation. Three-dimensional (3D) classification revealed three states with different conformations at the seam and maps were refined to resolutions of 4–6 Å. These states were termed WT-1, WT-2A, and WT-2B and populated to 15%, 43%, and 20%, respectively (Figures 5A and S4B). WT-1 and WT-2A are largely similar to states KC-1 and KC-2A of ClpB-K476C, whereas WT-2B is reminiscent of ClpB-K476C state 2B. A structural equivalent to ClpB-K476C state 3 is missing in the ClpB-WT population.

**Figure 6:**
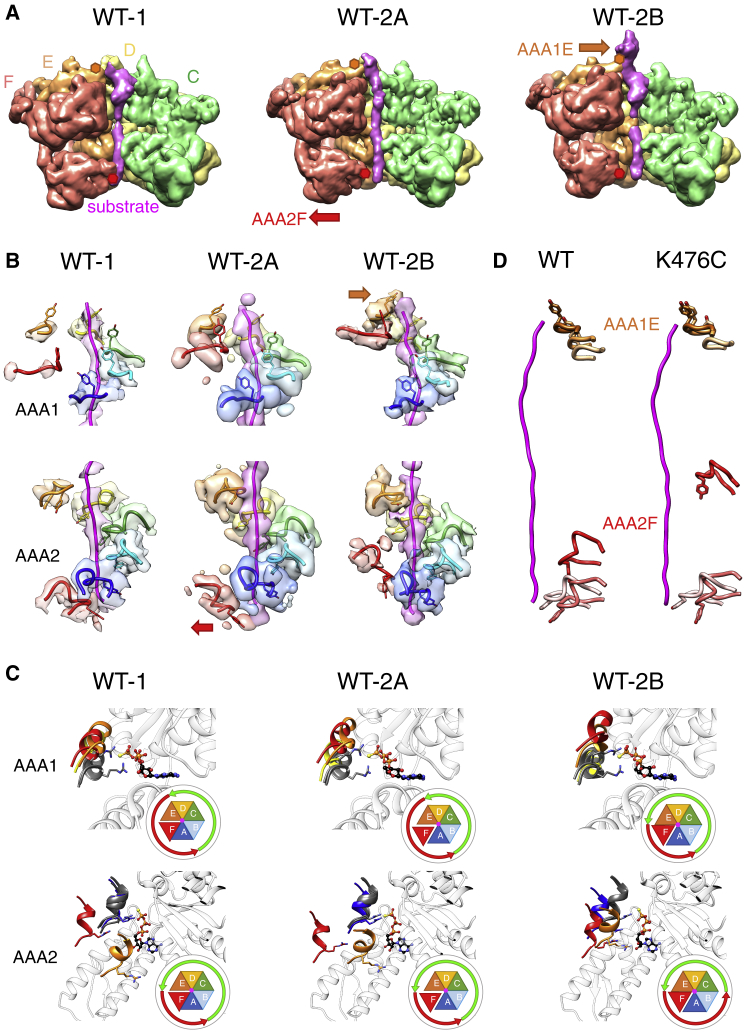
Overview of Substrate-Bound ClpB-DWB, Pore Loop Movements and Arginine Finger Contacts (A) Views of the cryo-EM maps of the three states of substrate-bound ClpB-DWB. Densities of protomers A and B were removed to show conformational changes in protomers AAA1E and AAA2F, highlighted by orange and red arrows, respectively. Orange/red hexagons show the position of moving pore loops. (B) Interactions of ClpB-WT pore loops of the AAA1 (upper panel) and AAA2 (lower panel) rings with the substrate. The pore loop AAA2F (red) dissociates from the substrate in WT-2A. The pore loop AAA1E (orange) binds substrate in WT-2B. (C) Activity states of ClpB-WT AAA1 (upper panels) AAA2 (lower panels) domains. All protomers were aligned to the large AAA1 (AAA2) domain of protomer C to compare engagement of the arginine fingers with neighboring subunits. Arginine fingers of AAA1A-C and AAA2B-D are shown as grey ribbons and interact with the γ-phosphate of ATP bound at the active site of a neighboring subunit in all three states. Activity states of AAA1/2 protomers are indicated by green (active) and red (inactive) arrows. (D) Comparison of ClpB-WT and ClpB-K476C pore loop positions of AAA1E and AAA2F for states WT-1, WT-2A to WT-2B (pale to bright colors) and for states KC-1, KC-2 to KC-3 (pale to bright colors).

In all three ClpB-WT states all M-domains are fully visible and enclose the AAA1 ring, in agreement with previous findings (Figures 6A and 6B; Deville et al., 2017). They adopt a horizontal “repressed” conformation, with head-to-tail contacts around the ring. Full enclosure of the ClpB-WT AAA1 ring by the M-domains might restrict the mobility of the entire ClpB hexamer, thereby impeding structural changes required for subunit cycling and high threading activity.

A continuous M-domain belt is not observed in ClpB-K476C, in which the M-domains show conformational heterogeneity with density for both horizontal “repressed” and tilted “derepressed” conformations (Figures 6A and 6B). Forty percent of ClpB-K476C particles have all six M-domains in a tilted conformation with only their motifs 1 but not their motifs 2 visible, because of high flexibility. Sixty percent of particles have M-domains of protomers D–F in a horizontal, docked conformation, while M-domains of protomers A–C are detached (Figures 6A and 6B). Detached M-domains are always associated with active ClpB-K476C protomers. The diversity of M-domain conformations in ClpB-K476C indicates increased structural flexibility, in agreement with previous biochemical analysis (Oguchi et al., 2012). The ratio of dissociated to attached M-domain conformations does not differ among the ClpB-K476C structural states. Notably, the partial M-domain docking in ClpB-WT (without substrate) and ClpB-K476C (with substrate) always includes the seam subunit AAA1F.

We next analyzed whether the complete docking of M-domains in ClpB-WT influences the conformational states and cycling of AAA domains by comparing ClpB-WT and ClpB-K476C structural states. We noticed that five ATPase sites are always inactive in ClpB-WT compared with four in ClpB-K476C. A major and mechanistically important difference between ClpB-WT and ClpB-K476C represents the loss of coordinated intra-ring subunit cycling in ClpB-WT. Although we observe activation and inactivation events (WT-2B: AAA1E activation, AAA2B inactivation) in ClpB-WT, these are not coupled within the same ring but between the rings, indicating differences in ring allostery between ClpB-WT and ClpB-K476C. Thus, inactivation of AAA2B in state WT-2B is not coupled to AAA2F activation as seen in ClpB-K476C (state KC-3) but to activation of AAA1E (Figure 5D). Furthermore, subunit activation (AAA1E) in the AAA1 ring and subunit inactivation (AAA2B) in the AAA2 ring occur simultaneously in ClpB-WT rather than offset, as observed for ClpB-K476C. Also, substrate engagement by the AAA1E pore loop no longer coincides with simultaneous dissociation of the AAA2F pore loop (Figure 5B). Activation of AAA2F, involving the dramatic rotation of AAA2F (44.5° upon conversion from KC-2 to KC-3), is not seen in ClpB-WT. This can be linked to the delayed activation of AAA1E that does not leave space for AAA2F rotation in ClpB-WT. As a result, the AAA2F pore loop remains at a lower position in WT-2B (11.7 Å upward movement) and does not move to the top of the spiral track as in KC-3 (33.8 Å upward movement) (Figures 5B and 5C; Video S2). Accordingly, the overall movement of the AAA2F seam subunit is more restricted in ClpB-WT (maximum root-mean-square deviation [RMSD] 5.9 Å) than in ClpB-K476C (maximum RMSD 8.1 Å).

Video S2 Comparison of AAA Domain Mobilities in ClpB- WT (Left) and ClpB- K476C (Right), Related to Figure 6. The movie shows morphs from WT-1/KC-1, to WT-2A/KC-2A, to WT-2B/KC-3.

We conclude that intra-ring and inter-ring communications differ in ClpB-WT and ClpB-K476C. Ordered cycling of AAA domains between different activity states and a large rearrangement of the AAA2 seam subunit and its pore loop are not observed in ClpB-WT. This suggests that the intra-ring coupling of ClpB-WT subunits is weaker than in ClpB-K476C, preventing the AAA2 motor ring of ClpB-WT from running in a sequential mode.

## Discussion

**Figure 7:**
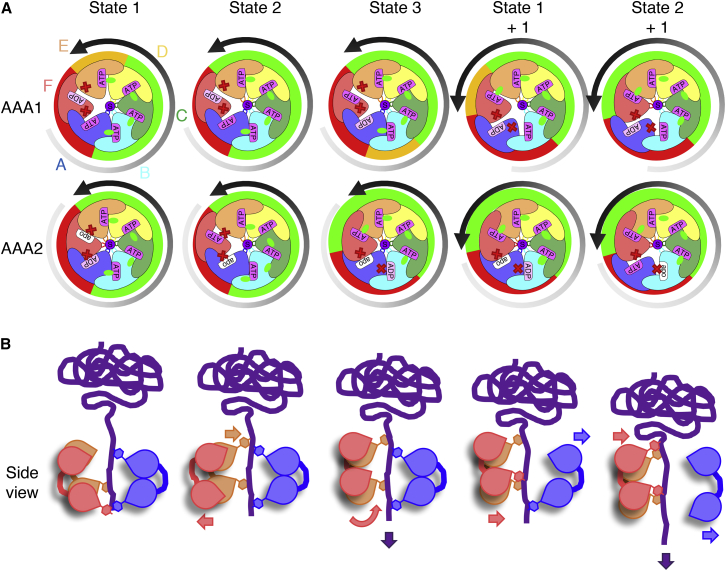
Coupled Sequential ATP Hydrolysis and Polypeptide Translocation upon ClpB Activation (A and B) Schematic representation of AAA1 and AAA2 rings (A) and side views of A, E, and F subunits bound to the substrate (B). Nucleotide states are shown in pink, pale pink, and white for ATP, ADP, and apo states, respectively. Engaged and detached arginine fingers are shown as green triangles and red crosses, respectively. Green, yellow, and red segments indicate activity states of AAA domains (active, intermediate, and inactive states, respectively). The gradient arrow shows pore loop position on the substrate from lowest in gray to highest in black. In this model, the AAA2 ring drives substrate threading, and the AAA1 ring follows conformational changes initiated in the AAA2 ring. Binding of ATP to apo AAA2F leads to its dissociation from the substrate and ADP release in AAA2A (state 2). ATP hydrolysis in AAA2B, triggered by the presence of apo AAA2A, allows detachment of the AAA2A arginine finger and repositioning of AAA2F to the top position (state 3). In the subsequent step (state 1 + 1) AAA2F contacts the substrate while AAA1A dissociates, resulting in the same conformation as state 1 but shifted by one protomer counterclockwise. This propels the substrate in discrete steps, ultimately causing the extraction of a polypeptide from a protein aggregate.

In this study we biochemically and structurally dissected the activation of a protein threading AAA+ machine with tandem AAA domains. We chose the ClpB disaggregase as model system because the signals triggering its activation are well defined: dissociation of inhibitory M-domain contacts and substrate binding. The comparison of ClpB-WT and derepressed ClpB-K476C enabled the mechanistic analysis of complexes exhibiting low and high ATPase and threading activities. We determined three structural snapshots of substrate-bound ClpB-K476C that likely constitute consecutive steps in a cycle in which ClpB activation triggers a sequential mechanism of ATP hydrolysis (Figure 7A). As our structural approach used the ATPase-deficient ClpB-K476C-DWB mutant, ClpB-K476C subunit cycling likely involves additional conformations that were not accessible here. We observe cycling of subunits between active and inactive states in both AAA1 and AAA2 rings, with changes being more pronounced in AAA2. Biochemical analysis of the ATPase mechanism of activated ClpB-K476C supports these findings. ATP hydrolysis by activated ClpB-K476C invokes a high degree of subunit coordination in a sequential rather than a concerted mechanism.

A sequential mechanism of ATP hydrolysis has previously been postulated for various protein threading AAA+ machines with single AAA domains, on the basis of single cryo-EM structures (Gates et al., 2017, Monroe et al., 2017, Puchades et al., 2017, Ripstein et al., 2017), with direct evidence from structures of the heterohexameric Rpt1-6 ATPase ring of the 26S proteasome and of archaeal PAN (de la Peña et al., 2018, Dong et al., 2019, Majumder et al., 2019). Our structural snapshots establish that the substrate threading mechanism is conserved for a homohexameric AAA+ protein with tandem AAA domains.

The activation of a sequential ATPase and threading mechanism involves several key features of the AAA2 ring. First, coupled activation and inactivation of ClpB AAA2 domains proceed in a counterclockwise direction as also observed in the 26S Rpt1-6 ring (de la Peña et al., 2018, Dong et al., 2019). Second, four active AAA2 domains are in almost identical states, and their ordered firing is controlled by the clockwise neighbor, which blocks hydrolysis when it has nucleotide bound and triggers hydrolysis in its apo state. Third, the cycling of AAA domains is directly linked to changes in the corresponding pore loop positions, providing the basis of mechanochemical coupling of ATP hydrolysis and force generation. The protomer contributing the pore loop at the lowest position of the spiral staircase of substrate interactions is always inactive. Activation of an AAA domain is linked to dramatic repositioning of its pore loop from the bottom to the top position. AAA domain activation either primes for (AAA2) or coincides with (AAA1) substrate engagement. ATP binding and hydrolysis events drive pore loop movements, which translocate the substrate in discrete, two-residue steps. Despite changes in the ClpB AAA1/2 domain activity states, the substrate is always bound by ten pore loops, ensuring high grip and tension force.

Our structures also provide insights into the division of labor and communication between the two AAA rings of this tandem AAA+ protein. Structural and biochemical data indicate that the AAA2 ring constitutes the main ATPase motor, whose activity is essential for ClpB activation. This is consistent with reports on the role of the AAA2 ring of ClpA, which is not controlled by M-domains and is thus constitutively activated (Kotamarthi et al., 2019, Kress et al., 2009). Furthermore, we observe offset cycling of AAA1 and AAA2 rings. Protomer activation in the AAA2 ring is shifted counterclockwise by one subunit relative to the AAA1 ring, which is required for ordered cycling of the AAA2 ring (Figure 7A). Accordingly, substrate interactions between AAA1 and AAA2 protomers are anti-correlated and substrate engagement in the AAA1 ring is directly coupled to substrate dissociation in the AAA2 ring leaving a constant number of ten AAA domains bound to the polypeptide substrate. This suggests that AAA1 and AAA2 domains work sequentially rather than simultaneously in the rope-climbing mechanism of substrate threading, ensuring sufficient grip while ClpB is moving along the substrate (Figure 7B; Video S1). This finding differs from a model of substrate threading by the yeast Hsp104 disaggregase proposing simultaneous action by AAA1 and AAA2 domains of the same protomer (Gates et al., 2017). Our biochemical analysis of ClpB as well as earlier work on Hsp104 (Franzmann et al., 2011) also indicate offset activities of AAA1 and AAA2 rings as well. This argues against concurrent action and suggests that the communication between AAA1 and AAA2 rings established here for ClpB also characterizes the yeast disaggregase.

Previous findings indicated that ClpB-WT does not thread with high processivity (Li et al., 2015) and cannot continuously apply high unfolding forces in contrast to derepressed M-domain mutants (Haslberger et al., 2008). Cryo-EM structures of substrate-bound ClpB-WT show a more restricted range of movement than ClpB-K476C, altered intra- and inter-ring subunit coupling, and fewer active nucleotide sites, providing a rationale for lower activity. There is no ClpB-WT structure similar to the ClpB-K476C KC-3 state. We cannot exclude that a very minor population of ClpB-WT adopts this conformation. This could permit sequential ATP hydrolysis by ClpB-WT in AAA2, but subunit cycling and therefore substrate threading would likely be restricted. Thus, ClpB-WT does not display the coupled activation and inactivation of AAA2 domains and accompanying dramatic pore loop movement of the AAA2 seam protomer. Accordingly, analysis of the substrate-stimulated ATPase activity of ClpB-WT revealed weaker subunit coordination than in ClpB-K476C. We infer that full activation of ClpB-WT is likely restricted to initial substrate engagement. This regulation allows high ClpB activity upon Hsp70-mediated recruitment to aggregates, while ensuring return to the low-activity mode upon loss of Hsp70 interaction. This mechanism protects cellular proteins from the cytotoxic activity characteristic of constitutively active M-domain mutants (Lipińska et al., 2013, Oguchi et al., 2012, Schirmer et al., 2004).

The differences in substrate-stimulated ATPase mechanism between ClpB-WT and ClpB-K476C correlate with the docking states of the repressive M-domains, the key structural element regulating ClpB activity (Haslberger et al., 2007, Oguchi et al., 2012, Rosenzweig et al., 2013). Substrate engagement by ClpB-WT causes full enclosure of the AAA1 ring by all M-domains in a constrained, horizontal conformation. In contrast, M-domains of substrate-bound ClpB-K476C are either partially or fully displaced from the AAA1 ring. This illustrates that the M-domain docking state controls ClpB ATPase activity, in agreement with previous findings (Carroni et al., 2014). Why does M-domain undocking allow ClpB to run in a sequential mode hydrolyzing ATP at high rates? In all cryo-EM structures, M-domains interact only with the AAA1 ring, arguing against direct signaling to AAA2. We speculate that full docking of M-domains to the AAA1 ring will strongly restrict its mobility, in particular at the dynamic hotspot, the seam subunit. This is in agreement with biochemical analysis showing lower flexibility of AAA domains in *Chaetomium thermophilum* Hsp104-WT than in a derepressed Hsp104-K494A M-domain mutant (equivalent to ClpB-K476C) (Heuck et al., 2016). When partial docking of M-domains is observed for ClpB-K476C (with substrate) (Figure 6A) or ClpB-WT (without substrate) (Deville et al., 2017), the interaction sites always include the seam subunit, implying that this site is particularly under M-domain constraint. We speculate that encircling of the AAA1 ring by docked M-domains slows down the conformational dynamics of the AAA1 ring, thereby hindering the coupled continuous cycling of the motor AAA2 domains.

In summary, we provide structural and biochemical evidence for regulated initiation of sequential ATPase and threading by a tandem AAA+ machine. The activation mechanism involves dissociation of repressive M-domains that likely function as mechanical brakes. This model explains how AAA+ protein activity can be tightly controlled to run in low- and high-ATPase and threading modes.

## STAR★Methods

### Key Resources Table

**Table:** 

REAGENT or RESOURCE	SOURCE	IDENTIFIER
**Bacterial and Virus Strains**		

*E. coli* XL1 blue	Agilent	200249
*E. coli* BL21 Rosetta	Merck/Novagen	70954

**Chemicals, Peptides, and Recombinant Proteins**		

Protease inhibitors	Roche	05056489001
FITC-casein	Sigma	C3777
Pyruvate kinase	Roche	10128163001
ATP	Sigma	A26209
ATPγS	Roche	11162306001
L-Malate Dehydrogenase	Sigma	10127256001
α-casein	Sigma	C6780
NADH	Sigma	43420
Phosphoenolpyruvate	Sigma	P7127
DMSO	Sigma	276855
Pyruvate kinase / Lactate Dehydrogenase mix (ATPase assay)	Sigma	P0294
ClpB wild type and mutants	Bukau Lab	N/A
IPTG	Roth	3216,4
Phusion DNA polymerase	Thermo Fisher Scientific	F530L
T4 DNA Ligase	Thermo Fisher Scientific	EL0011

**Critical Commercial Assays**		

GenElute PCR Clean-Up Kit	Sigma	NA1020
GenElute HP Plasmid Miniprep Kit	Sigma	NA0160
GenElute Gel Extraction Kit	Sigma	NA1111

**Oligonucleotides**		

ClpB E279A FP CATCCTATTTATCGACGCGTTACATA CCATGGTC	This paper	N/A
ClpB E279A RP GACCATGGTATGTAACGCGTCGATA AATAGGATG	This paper	N/A
ClpB E678A FP CCTGCTGGATGCGGTGGAAAAAGCG	This paper	N/A
ClpB E678A RP CGCTTTTTCCACCGCATCCAGCA G	This paper	N/A
ClpB Y251A FP GGGGCGAAAGCGCGCGGTGA	This paper	N/A
ClpB Y251A RP TCACCGCGCGCTTTCGCCCC	This paper	N/A
ClpB Y653A FP CGCCTCCGGGAGCGGTCGGTTA	This paper	N/A
ClpB Y653A RP TAACCGACCGCTCCCGGAGGCG	This paper	N/A
ClpB K476C FP GTACGCAGACCATTTGCGCGGAACTG	This paper	N/A
ClpB K476C RP CAGTTCCGCGCAAATGGTCTGCGTAC	This paper	N/A

**Recombinant DNA**		

pET24a-ClpB (& derivatives)	Bukau lab; this paper	N/A

**Software and Algorithms**		

Prism 6	https://www.graphpad.com	N/A
Origin	https://www.originlab.com	
RELION v2.1	Scheres, 2012	https://www2.mrc-lmb.cam.ac.uk/relion/index.php?title=Main_Page
MotionCor2	Zheng et al., 2017	http://msg.ucsf.edu/em/software/motioncor2.html
cryoSPARC	Punjani et al., 2017	https://www.nature.com/articles/nmeth.4169
Coot v0.8.8	Emsley et al., 2010	http://scripts.iucr.org/cgi-bin/paper?S0907444910007493
PHENIX v1.13	Adams et al., 2010	http://www.phenix-online.org/
UCSF Chimera	UCSF Resource for Biocomputing, Visualization, and Informatics	https://www.cgl.ucsf.edu/chimera/

**Other**		

Quantifoil UltrAuFoil R 1.2/1.3 electron microscopy grids	Quantifoil Micro Tools	R 1.2/1.3
Graphene oxide 2mg/mL dispersion in H2O	Sigma	763705

**Deposited data**		

ClpB-DWB-K476C bound to casein and ATPγS M-domain conformation 1 cryoEM map	This paper	EMDB: 4622
ClpB-DWB-K476C bound to casein and ATPγS M-domain conformation 2 cryoEM map	This paper	EMDB: 4623
ClpB-DWB-K476C bound to casein and ATPγS state KC-1 cryoEM map	This paper	EMDB: 4624
ClpB-DWB-K476C bound to casein and ATPγS state KC-2 cryoEM map	This paper	EMDB: 4625
ClpB-DWB-K476C bound to casein and ATPγS state KC-2A cryoEM map	This paper	EMDB: 4626
ClpB-DWB-K476C bound to casein and ATPγS state KC-2B cryoEM map	This paper	EMDB: 4627
ClpB-DWB-K476C bound to casein and ATPγS state KC-3 cryoEM map	This paper	EMDB: 4621
ClpB-DWB bound to casein and ATPγS state WT-1 cryoEM map	This paper	EMDB: 4640
ClpB-DWB bound to casein and ATPγS state WT-2A cryoEM map	This paper	EMDB: 4641
ClpB-DWB bound to casein and ATPγS state WT-2B cryoEM map	This paper	EMDB: 4642
ClpB-DWB-K476C bound to casein and ATPγS state KC-1 atomic coordinates	This paper	PDB: 6QS6
ClpB-DWB-K476C bound to casein and ATPγS state KC-2A atomic coordinates	This paper	PDB: 6QS7
ClpB-DWB-K476C bound to casein and ATPγS state KC-2B atomic coordinates	This paper	PDB: 6QS8
ClpB-DWB-K476C bound to casein and ATPγS state KC-3 atomic coordinates	This paper	PDB: 6QS4
ClpB-DWB bound to casein and ATPγS state WT-1 atomic coordinates	This paper	PDB: 6RN2
ClpB-DWB bound to casein and ATPγS state WT-2A atomic coordinates	This paper	PDB: 6RN3
ClpB-DWB bound to casein and ATPγS state WT-2B atomic coordinates	This paper	PDB: 6RN4

### Contact for Reagent and Resource Sharing

Further information and requests for resources and reagents should be directed to and will be fulfilled by the Lead Contact, Helen Saibil (h.saibil@mail.cryst.bbk.ac.uk).

### Experimental Model and Subject Details

*E. coli* XL1 blue and *E. coli* BL21 Rosetta strains were used in this study. Cells were grown at 30°C or 37°C in LB medium.

### Method Details

#### Construction and purification of ClpB variants

ClpB was amplified by PCR and inserted into pDS56 and verified by sequencing. Mutant derivatives of *clpB* were generated by PCR mutagenesis and standard cloning techniques in pDS56 and were verified by sequencing. ClpB was purified after overproduction from *E. coli* Δ*clpB::kan* cells. ClpB wild-type and mutant variants were purified using Ni-IDA (Macherey-Nagel) and size exclusion chromatography (Superdex S200, Amersham) following standard protocols. Purifications of DnaK, DnaJ, GrpE, Luciferase and Casein-YFP were performed as described previously (Haslberger et al., 2008, Oguchi et al., 2012, Seyffer et al., 2012). Pyruvate kinase of rabbit muscle and Malate Dehydrogenase of pig heart muscle were purchased from Sigma. Protein concentrations were determined with the Bio-Rad Bradford assay.

#### ATPase assay

ClpB ATPase activities were determined in Reaction buffer (50 mM Tris pH 7.5, 25 mM KCl, 20 mM MgCl_2_, 2 mM DTT) in the absence or presence of substrate (10 μM casein) using a NADH-coupled colorimetric assay (Sigma) by measuring the decrease of NADH absorption at 340 nm in a BMG Labtech FLUOstar Omega plate reader. ClpB wild-type and variants were typically used at 0.5 μM except for K476C (0.15 μM in presence of casein) and E279A or K476C/E279A (0.25 μM in presence of casein). ATPase activities were derived from the linear decrease of NADH absorbance and K_0.5_, v_max_ and n_Hill_ values were determined by fitting the curves using nonlinear regression applying an allosteric sigmoidal model (Origin, Prism software).

#### MDH disaggregation

ClpB disaggregation activities were determined by following the disaggregation of heat-aggregated malate dehydrogenase (MDH: 1 μM, 30 min at 47°C) in Reaction buffer. Chaperones were used at the following concentrations: 1 μM ClpB (wild-type or derivatives), *E. coli* Hsp70 system: 1 μM DnaK, 0.2 μM DnaJ, 0.1 μM GrpE. Disaggregation reactions were performed in Reaction Buffer (50 mM Tris pH 7.5, 150 mM KCl, 20 mM MgCl_2_, 2 mM DTT) containing an ATP regenerating system (2 mM ATP, 3 mM phosphoenolpyruvate, 20 ng/μl Pyruvate Kinase) and the GroEL/GroES chaperone system (1 μM each) to couple MDH disaggregation with subsequent refolding. MDH reactivation was determined by determining MDH activity at different time points during disaggregation reaction. MDH activity was determined in 150 mM potassium phosphate buffer pH 7.6, 0.5 mM oxaloacetate, 0.28 mM NADH, 2 mM DTT by monitoring NADH oxidation at 340 nm with a NovaSpec Plus Photometer (GE Healthcare). Disaggregation rates were derived from the linear phase of MDH activity regain.

#### Anisotropy measurements

Binding of ClpB (WT or mutant) to FITC-casein (100 nM) was monitored by fluorescence anisotropy measurements using a BMG Biotech CLARIOstar platereader. Samples were incubated in Reaction buffer for 10 min at 30°C in presence of 2 mM ATP and polarization of FITC-casein was determined in black 384 well plates (excitation: 482 nm; emission: 530 nm, Target mP: 35). A sample containing FITC-casein only served as reference. K_d_ values were determined using nonlinear regression curve fitting (Prism software).

#### Sample preparation for cryo-electron microscopy

The ClpB:casein complex was formed by incubating ClpB hexamer with a 20-fold molar excess of casein, to maximize complex occupancy, for 10 minutes at room temperature in 25 mM Tris-HCl (pH = 7.4), 25 mM KCl, 10 mM MgCl2, 2mM ATPγS and 1mM DTT. For casein-bound Clp-DWB-K476C, the complex solution was diluted to 1.6 mg/ml of complex, applied on 1.2/1.3 300 mesh AuFoil grids (quantifoil) coated with graphene oxide and vitrified by plunge freezing in liquid ethane after blotting for 2 s using a Vitrobot (Thermofisher). For casein-bound ClpB WT, the complex solution was diluted to 8 mg/ml of complex, applied on 1.2/1.3 300 mesh AuFoil grids (quantifoil) and vitrified as above.

For casein-bound Clp-DWB-K476C in absence of substrate, the complex solution was diluted to ∼1 mg/ml, applied on lacey carbon grids with a carbon support, glow-discharged in presence of amylamine (quantifoil) and vitrified as above.

#### Cryo-electron microscopy image acquisition

Images were collected using EPU software on a Titan Krios transmission electron microscope (FEI) operating at 300 kV, equipped with a GatanK2 Summit direct electron detector and bioquantum energy filter with 20 eV slit. The defocus range was set between −1.5 and −3.5 um, and the total dose was ∼50 electrons/Å^2^. Pixel size was 1.055 Å/pixel for unbound casein-bound Clp-DWB-K476C and 1.043 Å/pixel for casein-bound ClpB-DWB and 1.4 Å/pixel for Clp-DWB-K476C without substrate.

#### Image processing

After initial sorting of the collected images, movie frames of each micrograph were aligned using MotionCor2 (Zheng et al., 2017) with 25 patches per image, and dose compensation was applied. The contrast transfer function was estimated with CTFFIND4 (Rohou and Grigorieff, 2015). Particles were picked without using a template using Gautomatch and extracted in Relion 2.1 (Scheres, 2012) with a 256x256 box size. The initial datasets were subjected to reference-free 2D classification in Cryosparc (Punjani et al., 2017) and poorly resolved class averages were removed. A consensus initial model was generated by *ab initio* reconstruction using stochastic gradient descent in Cryosparc. For ClpB-DWB-K476C in absence of substrate, *ab initio* model generation led to an open spiral and a closed ring conformation. Those two models were used for homogeneous 3D refinement of the corresponding particles in Cryosparc and post-processing yielded maps at an estimated 7-8 Å resolution (gold standard 0.143 FSC criterion). For processing of casein-bound ClpB-DWB-K476C, the Cryosparc initial closed ring model was used for homogeneous 3D refinement in Cryosparc and post-processing yielded a map at an estimated 3.4 Å resolution (gold standard 0.143 FSC criterion). The seam region of this map was poorly resolved and some of the middle domains showed density for multiple conformations. Heterogeneous refinement in Cryosparc with three classes revealed two conformations for the middle domains: (1) all tilted, 44% of the particles refining to 3.5 Å resolution (2) three tilted and three horizontal, 56% of the particles refining to 3.2 Å resolution. The AAA seam region was still poorly resolved in these maps. 3D classification with 10 classes was performed in Relion with an angular search restricted to 10° and 3D masking of the N-terminal domains and middle domains. Three out of ten classes showed different conformations and high-resolution features. Classes 4 and 9 yielded 3.9 Å resolution maps (states 1 and 4 respectively). Class 9 (state 4) had the 2nd AAA domain of protomer F (AAA2F) partly poorly resolved. Further classification was carried on with 3 classes, no alignment and a soft mask including only AAA2F. Class 9_2 yielded a 4.1 Å resolution map with a better resolved AAA2F. Class 10 yielded a 3.2 Å resolution map where AAA2F was still poorly resolved. Further classification was carried on with 6 classes, no alignment and a soft mask including AAA2F only. Two out of six classes showed different conformations for AAA2F with high resolution features. Classes 10_5 and 10_3 yielded 3.8 and 3.9Å resolution maps respectively (states 2 and 3 respectively). See also Figure S4A for details on data processing. A similar 3D classification scheme was applied to ClpB WT and 3 states were obtained (Figure S4B).

#### Model building and refinement

Because we obtained several maps with a variety of resolutions from 3.2 to 4.1 Å resolution, we first built a model for the highest resolution map and used it as a starting model for building into the other maps.

The crystal structure of ClpB (PDB: 4CIU), used as an initial model, was segmented into small and large sub-domains of AAA1 and AAA2 and middle domain. These segments were rigid body fitted into the density of class 10 protomer D using the Fit in map tool of Chimera (Pettersen et al., 2004). Missing loops and connecting regions were built in COOT (Emsley et al., 2010) and the model was further refined using COOT and Phenix (Adams et al., 2010). This subunit was copied and rigid body fitted into the densities for protomers B, C and E, which adopt a similar conformation to protomer D, with little difference between the 4 states. Further refinement of this tetramer was carried out in COOT and Phenix. For protomers A and F, sub-domains of the refined models were rigid body fitted in the density in Chimera. Connecting regions were built in COOT and the model was further refined using COOT and Phenix. The models of all other states were built from the model of state 2 as a starting point. For protomers displaying large conformational changes, sub-domains were rigid body fitted in the density in chimera before further refined using COOT and Phenix.

### Quantification and Statistical Analysis

ATPase activities were determined by calculating the linear decrease of NADH absorbance using Excel. The corresponding standard deviations were calculated by Excel. Hill coefficients of ATPase activities and their respective standard deviations were determined by Origin software. Binding affinities of ClpB to FITC-casein and their respective standard deviations were determined by Prism software. MDH disaggregation activities were determined by calculating the linear increase in MDH activity using Excel. Corresponding standard deviations were calculated by Excel. Quantification, statistical analysis and validation related to cryo-EM image processing are implemented in the software described in the image processing section of the methods details. The global resolution estimates of refined cryo-EM maps are based on the 0.143 cutoffs of the FSC between two half maps refined independently.

#### Data Availability

The cryo-EM maps and associated coordinates have been deposited in the EMDB and on the PDB: KC-1 (EMDB: 4624, PDB: 6QS6), KC-2 before AAA2F classification (EMDB: 4625), KC-2A (EMDB: 4626, PDB: 6QS7), KC-2B (EMDB: 4627, PDB: 6QS8), KC-3 (EMDB: 4621, PDB: 6QS4), KC M-domain conformation 1 MD-1 (EMDB: 4622), KC M-domain conformation 2 MD-2 (EMDB: 4623) WT-1 (EMDB: 4940, PDB: 6RN2), WT-2A (EMDB: 4941, PDB: 6RN3), WT-2B (EMDB: 4942, PDB: 6RN4).

## Supplemental Information

Document S1. Figures S1–S7 and Tables S1–S3

Document S2. Article plus Supplemental Information
