# Supplementary material for: Two-Step Activation Mechanism of the ClpB Disaggregase for Sequential Substrate Threading by the Main ATPase Motor
Source: Cell Rep. 2019 Jun 18;27(12):3433–3446.e4. doi: 10.1016/j.celrep.2019.05.075 (PMC6593972; doi:10.1016/j.celrep.2019.05.075)
Supplement: Document S1. Figures S1–S7 and Tables S1–S3 [file mmc1.pdf]

Cell Reports, Volume *27*

Supplemental Information

Two-Step Activation Mechanism of the ClpB Disaggregase for Sequential Substrate Threading by the Main ATPase Motor

Célia Deville, Kamila Franke, Axel Mogk, Bernd Bukau, and Helen R. Saibil

Two-step activation mechanism of the ClpB disaggregase for sequential substrate threading by the main ATPase motor

Célia Deville[1,2,*], Kamila Franke[3,*], Axel Mogk[3,4], Bernd Bukau[3,4], Helen R. Saibil[1,4]

[1] Department of Crystallography, Institute of Structural and Molecular Biology, Birkbeck, University of London, Malet Street, London WC1E 7HX, United Kingdom.
[2] present address: Department of Integrative Structural Biology, Institut de Génétique et de Biologie Moléculaire et Cellulaire, INSERM, U964, CNRS, UMR-7104, Université de Strasbourg, Illkirch–Graffenstaden, France.

[3] Center for Molecular Biology of University of Heidelberg (ZMBH) and German Cancer Research Center (DKFZ), DKFZ-ZMBH Alliance, Im Neuenheimer Feld 282, D-69120 Heidelberg, Germany.

[4] corresponding authors

Email:
h.saibil@mail.cryst.bbk.ac.uk (H. R. S.)
bukau@zmbh.uni-heidelberg.de (B.B)
a.mogk@zmbh.uni-heidelberg.de (A. M.)

* these authors equally contributed to this work

List of supplementary information

Supplementary figures 1 to 7

Supplementary tables 1 to 3

Supplementary movies 1 to 2

Figure S1

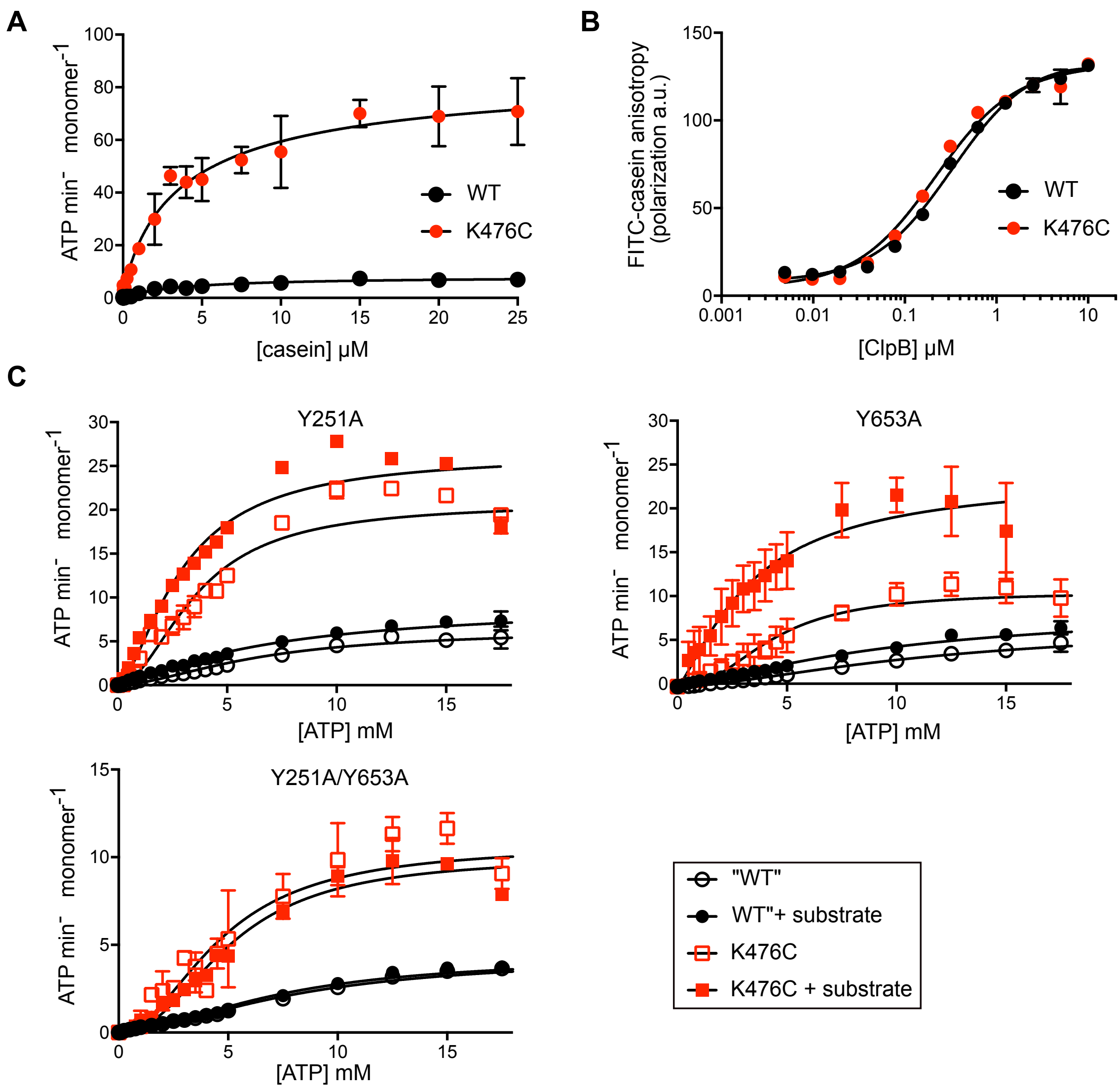

Figure S1. ATPase activities and substrate binding of ClpB-WT and mutants. Related to Figure 1. (A) ATPase activities of ClpB-WT and ClpB-K476C were determined in presence of increasing casein concentrations. (B) Binding of ATPase-deficient ClpB-E279A/E678A or ClpB-K476C/E279A/E678A to fluorescein-labeled FITC-casein was determined in presence of 2 mM ATP by anisotropy measurements. (C) ATPase activities of pore 1 and pore 2 mutants of ClpB-WT or ClpB-K476C were determined at the indicated ATP concentrations in absence and presence of substrate casein. Standard deviations are indicated; for some points error bars are shorter than the height of the symbol and are not depicted.

Figure S2

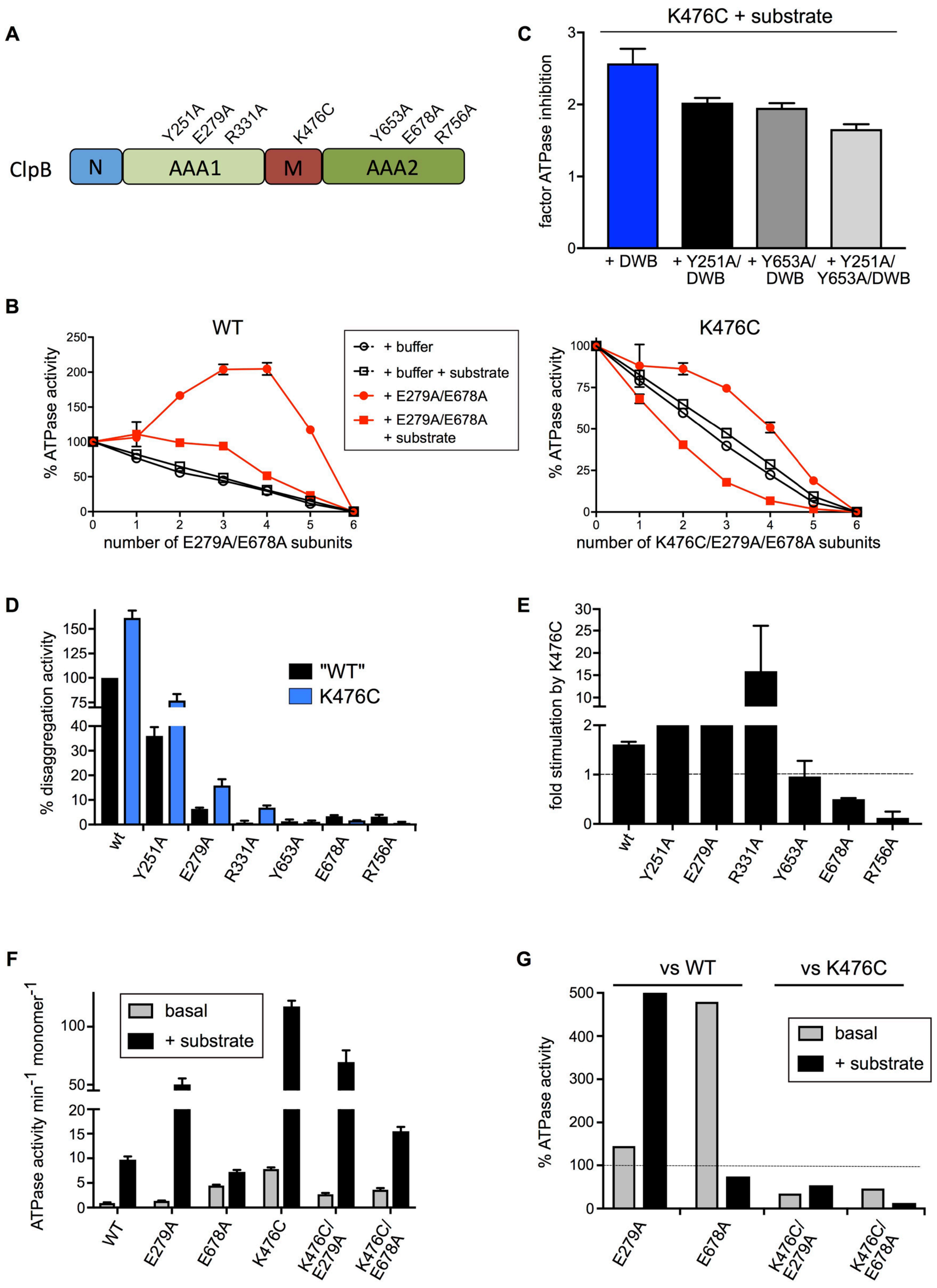
A
ClpB
N
AAA1
M
AAA2
Y251A
E279A
R331A
K476C
Y653A
E678A
R756A
B
WT
% ATPase activity
number of E279A/E678A subunits
+ buffer
+ buffer + substrate
+ E279A/E678A
+ E279A/E678A + substrate
K476C
number of K476C/E279A/E678A subunits
C
K476C + substrate
factor ATPase inhibition
+ DWB
+ Y251A/ DWB
+ Y653A/ DWB
+ Y251A/ Y653A/DWB
D
% disaggregation activity
"WT"
K476C
wt
Y251A
E279A
R331A
Y653A
E678A
R756A
E
fold stimulation by K476C
F
ATPase activity min-1 monomer-1
basal
+ substrate
WT
E279A
E678A
K476C
K476C/ E279A
K476C/ E678A
G
vs WT
vs K476C
% ATPase activity
basal
+ substrate

Figure S2. ATPase and disaggregation activities of ClpB WT and mutants. Related to Figure 1. (A) ClpB domain organization and diverse mutants affecting substrate binding and threading (Y251A, Y653A) or ATP hydrolysis (E279A, R331A, E678A, R756A). (B) ATPase activities of mixtures of ClpB-WT or ClpB-K476C and corresponding E279A/E678A mutant subunits that bind but cannot hydrolyze ATP were determined in the absence and presence of substrate. The ATPase activities of ClpB-WT or ClpB-K476C dilutions corresponding to their concentrations in the mixes were determined as reference. The ATPase activities of non-diluted ClpB-WT or ClpB-K476C were set to 100%. Mixing ratios are indicated as number of E279A/E678A mutant subunits. (C) ATPase activities of 1:1 mixtures of ClpB-K476C and its ATPase deficient E279A/E678A (DWB) or pore mutant derivatives were determined in presence of substrate and compared to non-mixed ClpB-K476C. The factor of ATPase inhibition by mutant subunit incorporation was determined (factor 1: no change). (D) MDH disaggregation activities of ClpB wild type (WT) and ClpB-K476C and Walker B (E279A, E678A), Arginine finger (R331A, R756A) and pore loop (Y251A, Y653A) mutant derivatives of AAA-1 or AAA-2 domains were determined. The activity of ClpB-WT was set to 100%. (E) The relative increase in MDH disaggregation activities for the indicated mutant protein upon relief of M-domain repression (K476C) was determined. (F) ATPase activities of ClpB wild type (WT) and ClpB-K476C derivatives that can only hydrolyse ATP at AAA-2 (E279A) or AAA-1 (E678A) were determined in absence (basal) and presence of casein (+ substrate). Standard deviations are indicated; for some points error bars are shorter than the height of the symbol and are not depicted. (G) Changes in ATPase activities in ClpB mutants that can hydrolyse ATP only at either AAA-2 (E279A) or AAA-1 (E678A). Basal and substrate-stimulated ATPase activities of ClpB WT or ClpB-K476C were set to 100%.

Figure S3

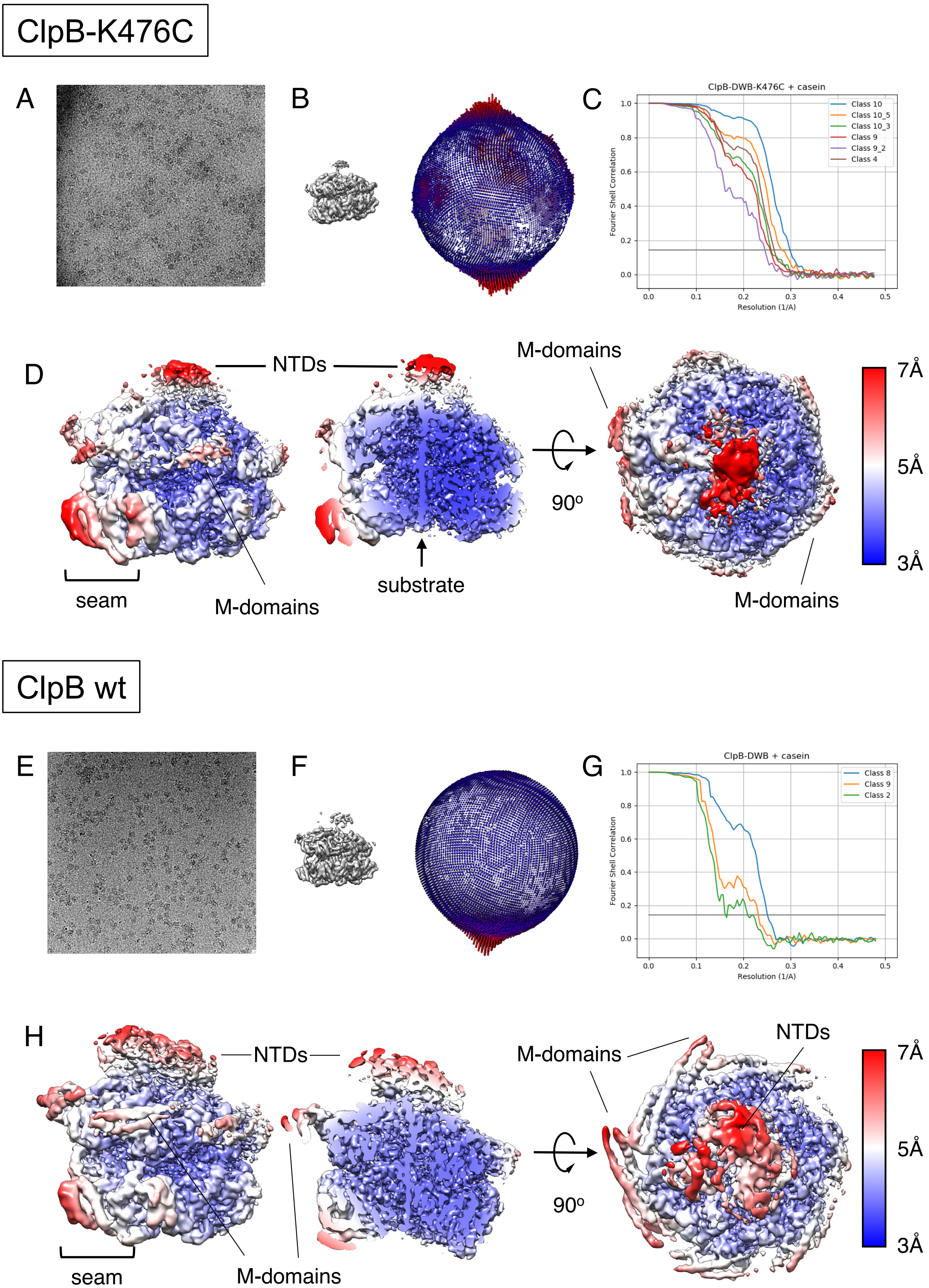
ClpB-K476C
A
B
C
ClpB-DWB-K476C + casein
Class 10
Class 10_5
Class 10_3
Class 9
Class 9_2
Class 4
Fourier Shell Correlation
Resolution (1/A)
D
NTDs
M-domains
90o
7Å
5Å
3Å
seam
M-domains
substrate
M-domains
ClpB wt
E
F
G
ClpB-DWB + casein
Class 8
Class 9
Class 2
Fourier Shell Correlation
Resolution (1/A)
H
NTDs
M-domains
NTDs
90o
7Å
5Å
3Å
seam
M-domains

Figure S3. Analysis of substrate-bound ClpB-DWB-K476C and ClpB-DWB cryo-EM data. Related to STAR methods. (A) Representative micrograph of the ClpB-DWB-K476C:casein complex in the presence of ATPγS, on graphene oxide support. (B) Angular distribution of the particles in the final reconstruction for 10_5. There are preferential orientations for top views as well as some side views. Other states display similar angular distributions. (C) Gold standard Fourier shell correlation (FSC) curves for different ClpB-DWB-K476C:casein reconstructions. (D) Plot of the local resolution on the surface of the class 10_5 density map from a side view (left), cut view (middle) and top view (right). The resolution is lower for N-terminal domains (NTDs), the partly visible M-domains and the AAA domains of the A and F seam protomers. (E) Representative micrograph of the ClpB-DWB:casein complex in the presence of ATPγS. (F) Angular distribution of the particles in the final reconstruction for class 8. There are preferential orientations for top views as well as some side views. Other states display similar angular distributions. (G) Gold standard Fourier shell correlation (FSC) curves for different ClpB-DWB:casein reconstructions. (H) Plot of the local resolution on the surface of the class 8 density map from a side view (left), cut view (middle) and top view (right). The resolution is lower for the N-terminal domains (NTDs), M-domains and the AAA domains of A and F seam protomers.

Figure S4

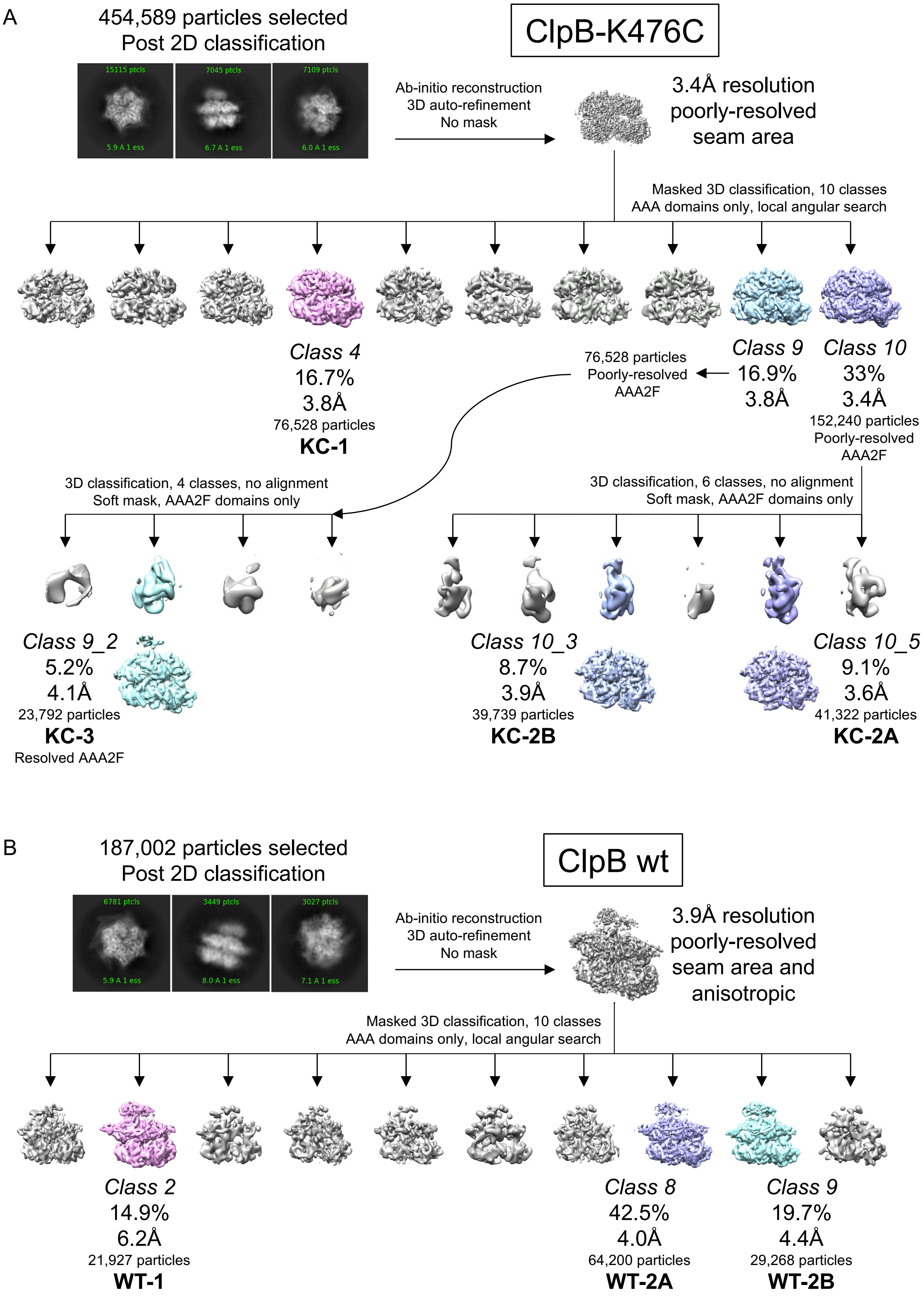

A
454,589 particles selected
Post 2D classification
15115 ptcls
7045 ptcls
7109 ptcls
5.9 A 1 ess
6.7 A 1 ess
6.0 A 1 ess
ClpB-K476C
Ab-initio reconstruction
3D auto-refinement
No mask
3.4Å resolution
poorly-resolved
seam area
Masked 3D classification, 10 classes
AAA domains only, local angular search
Class 4
16.7%
3.8Å
76,528 particles
KC-1
76,528 particles
Poorly-resolved
AAA2F
Class 9
16.9%
3.8Å
Class 10
33%
3.4Å
152,240 particles
Poorly-resolved
AAA2F
3D classification, 4 classes, no alignment
Soft mask, AAA2F domains only
3D classification, 6 classes, no alignment
Soft mask, AAA2F domains only
Class 9_2
5.2%
4.1Å
23,792 particles
KC-3
Resolved AAA2F
Class 10_3
8.7%
3.9Å
39,739 particles
KC-2B
Class 10_5
9.1%
3.6Å
41,322 particles
KC-2A
B
187,002 particles selected
Post 2D classification
6781 ptcls
3449 ptcls
3027 ptcls
5.9 A 1 ess
8.0 A 1 ess
7.1 A 1 ess
ClpB wt
Ab-initio reconstruction
3D auto-refinement
No mask
3.9Å resolution
poorly-resolved
seam area and
anisotropic
Masked 3D classification, 10 classes
AAA domains only, local angular search
Class 2
14.9%
6.2Å
21,927 particles
WT-1
Class 8
42.5%
4.0Å
64,200 particles
WT-2A
Class 9
19.7%
4.4Å
29,268 particles
WT-2B

Figure S4. Classification workflow used for data processing of the ClpB-DWB-K476C:casein (A) and ClpB-DWB:casein (B) complex structures. Related to STAR methods. The classes selected for further refinement are shown in color. (A): pink for class 4 yielding state KC-1, purple for classes 10, 10_3 and 10_5 yielding states KC-2A and KC-2B and blue for class 9 and 9_2 yielding state KC-3. (B): pink for class 2 yielding state WT-1, purple for class 8 yielding state WT-2A and blue for class 9 yielding state WT-2B.

Figure S5

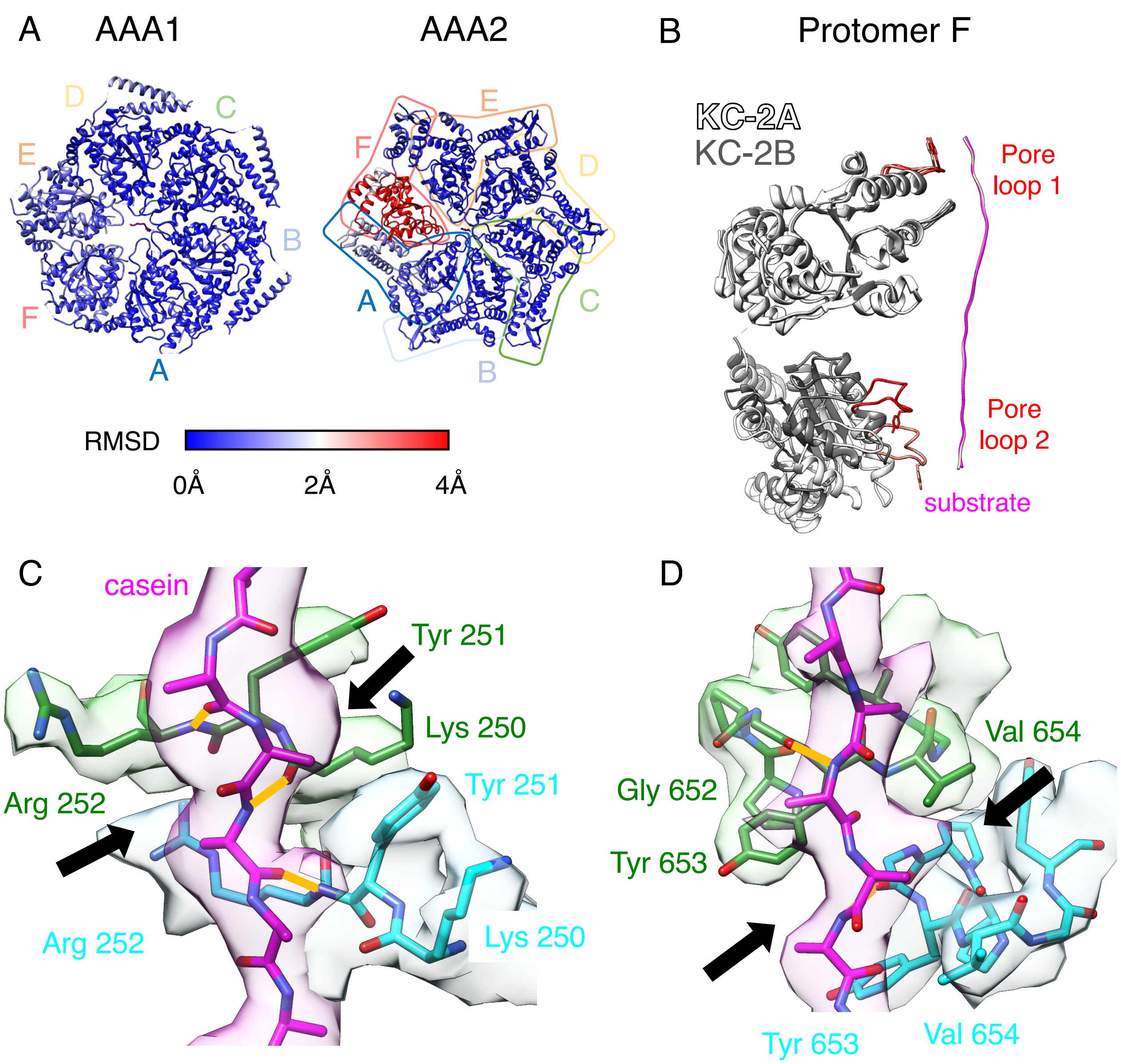

Figure S5. Comparison of states KC-2A and KC-2B and details of ClpB-K476C pore loop - substrate interactions. Related to Figures 2 and 3. (A) RMSD between the KC-2A and KC-2B states is displayed on the KC-2A atomic model. Transitions from identical to different conformations are colored from blue to red. (B) Superimposed atomic models of protomer F and substrate casein of KC-2A (white) and KC-2B (grey). The pore loops are shown in orange (KC-2A) and red (KC-2B). (C,D) Overall organisation of ClpB-K476C pore loops bound to the substrate modelled as a poly-alanine chain (shown for KC-2A). The pore loops including

conserved tyrosine residues form a spiral track of interactions and bind to the extended substrate in steps of two residues. Close-up of the ClpB-K476C:casein interactions in AAA1 (C) and AAA2 (D), shown for protomers B and C. The backbones of Lys250 and Arg252 in AAA1 and of Gly652 in AAA2 form hydrogen bonds with the backbone of the substrate. Pockets that accommodate the substrate side chains are shown with black arrows. They are formed by tyrosine side chains, pointing up in AAA1 and down in AAA2, on the one hand and by stacked Arg252 in AAA1 and stacked Val654 in AAA2 on the other hand.

Figure S6

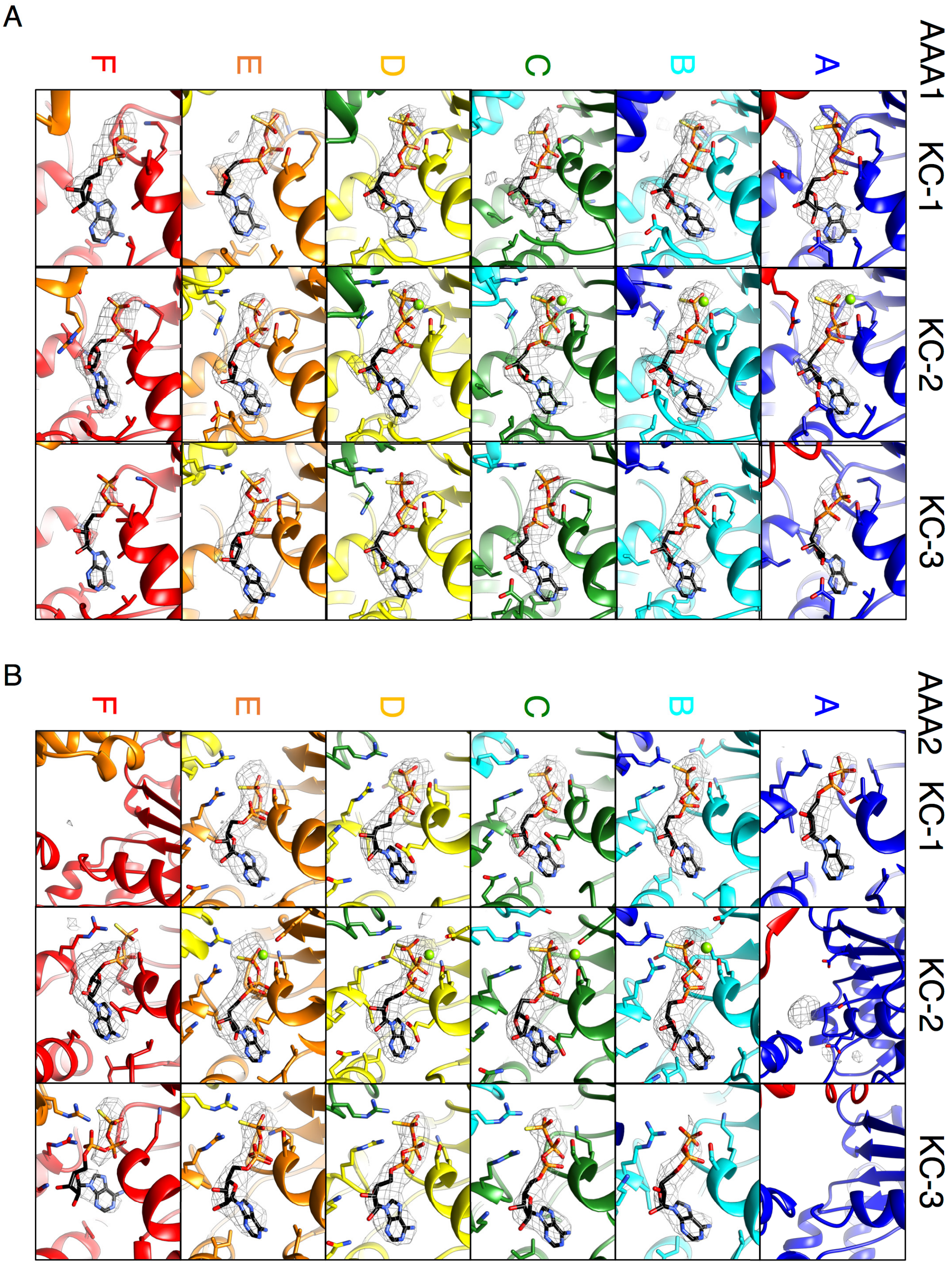

Figure S6. Nucleotide densities. Related to figure 3. (A) AAA1 domains and (B) AAA2 domains, for KC-1, KC-2 and KC-3 states. Densities are shown at the same threshold for each state.

Figure S7

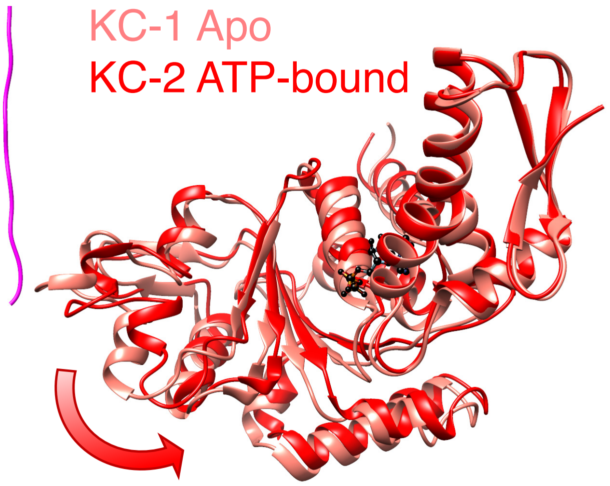

Figure S7. Dissociation of AAA2F upon conversion from KC-1 to KC-2. Related to Figure 4. Apo AAA2F is bound to the substrate in KC-1 (in salmon color). Binding of ATP induces closing of the hinge between the small and large domains of AAA2F, thus triggering detachment of AAA2F from the substrate in KC-2 (in red).

Table S1: Steady state ATPase activities of ClpB wild type (WT) and mutants in the absence and presence of substrate (+ casein). Related to Figure 1.

ClpB variant	v_{max} (min^{-1})	$K_{0.5}$ (mM)	n_{Hill}
WT	8.8 ± 0.5	8,7 ± 0.5	2,5 ± 0.2
WT+ casein	25.9 ± 1.2	4,3 ± 0.3	1,5 ± 0.1
Y251A	5.53 ± 0,4	5.29 ± 0.23	2.12 ± 0.18
Y251A + casein	7.33 ± 0,5	4.51 ± 0.2	1.69 ± 0.21
Y653A	5.01 ± 0,4	7.52 ± 0.27	2.18 ± 0.14
Y653A + casein	6.72 ± 0,5	6.2 ± 0.3	1.85 ± 0.15
Y251A/Y653A	3.65 ± 0,3	6.27 ± 0.27	2.14 ± 0.17
Y251A/Y653A + casein	3.74 ± 0,3	5.92 ± 0.25	2.26 ± 0.29
K476C	37.0 ±1.3	5,1 ± 0.2	1,9 ± 0.1
K476C +casein	130.2 ± 6.0	1,4 ± 0.1	1,3 ± 0.1
K476C/Y251A	22.43 ± 1,1	3.93 ± 0.21	1.91 ± 0.2
K476C/Y251A + casein	27.78 ± 1,2	3,14 ± 0.14	1,62 ± 0.13
K476C/Y653A	11.68 ± 1,7	4.68 ± 0.19	2.16 ± 0.19
K476C/Y653A + casein	21.85 ± 1,9	2,86 ± 0.16	1,57 ± 0.16
K476C/Y251A/Y653A	11.64 ± 0,7	5.04 ± 0.34	2.09 ± 0.29
K476C/Y251A/Y653A + casein	9.78 ± 0,6	4.89 ± 0.17	2,47 ± 0.21

Table S2: Details of cryo-electron microscopy image acquisition. Related to STAR Methods.

Complex	ClpB-DWB-K476C + casein + ATPγS	ClpB-DWB + casein +ATPγS
Microscope	Titan Krios	
Voltage	300kV	
Camera	K2 summit (Gatan)	
Pixel size (Å)	1.05	1.04
Total dose (electrons/Å^2)	50	50
Number of frames	50	42
Defocus range	-1.5 to -3.5 um	-1.5 to -3.5 um
Number of movies	6060	1687
Nb of particles picked	875,861	290,830
Nb of particles refined	454,589	187,002
Grid type	AuFoil 1.2/1.3, 300 mesh, GO coated	AuFoil 1.2/1.3, 300 mesh

Table S3: Details of cryo-EM density maps. Related to STAR Methods

State	Class	0.143 FSC cutoff	Number of particles	EMDB code	PDB code
**ClpB-DWB-K476C:casein**					
KC-1	4	3.78 Å	76,528	4624	6QS6
KC-2	10	3.36 Å	152,240	4625	
KC-2A	10_5	3.60 Å	41,322	4626	6QS7
KC-2B	10_3	3.89 Å	39,739	4627	6QS8
	9	3.93 Å	76,528		
KC-3	9_2	4.09 Å	23,792	4621	6QS4
MD-1		3.68Å	224,759	4622	
MD-2		3.53Å	165,200	4623	
**ClpB-DWB:casein**					
WT-1	2	6.21Å	21,927	4940	6RN2
WT-2A	8	4.04Å	64,200	4941	6RN3
WT-2B	9	4.38Å	29,268	4942	6RN4

Movie S1. Movie1wholePDB.mov

Movie S2. Movie2WTvsKC.mov
